# Incidence of Coronary Obstruction During Aortic Valve Implantation: Meta-Analysis and Mixt-Treatment Comparison of Self-Expandable Versus Balloon-Expandable Valve Prostheses

**DOI:** 10.31083/RCM36208

**Published:** 2025-07-29

**Authors:** Yu Fei Wang, Zai Qiang Liu, Xiao Teng Ma, Li Xia Yang, Zhi Jian Wang, Yu Jie Zhou

**Affiliations:** ^1^Cardiology Division, Beijing Jishuitan Hospital, Capital Medical University, 100035 Beijing, China; ^2^Department of Cardiology, Beijing Anzhen Hospital, Capital Medical University, 100029 Beijing, China; ^3^Beijing Institute of Heart Lung and Blood Vessel Disease, The Key Laboratory of Remodeling-related Cardiovascular Disease, Ministry of Education, 100029 Beijing, China

**Keywords:** transcatheter aortic valve replacement, self-expanding valves, balloon-expandable valves, surgical aortic valve replacement, coronary obstruction

## Abstract

**Background:**

Recently, the transcatheter aortic valve replacement (TAVR) indications have expanded; meanwhile, valve systems have continuously evolved and improved. However, coronary occlusion (CO), a rare but catastrophic consequence of TAVR surgery, limits the expansion of indications for TAVR. Moreover, comparisons between different systems remain scarce. This study aimed to evaluate the incidence of CO associated with TAVR, specifically comparing self-expanding valves (SEVs) and balloon-expandable valves (BEVs), and further assess the safety profile of these valve subtypes.

**Methods:**

The primary outcome of interest was the incidence of CO during TAVR using BEVs or SEVs. Electronic databases were searched from January 2009 to June 2023, and this study included randomized controlled trials, observational studies, and propensity pair-matched studies. Heterogeneity and inter-study variance were assessed using Cochran’s Q, I^2^, and τ^2^ (Sidik–Jonkman estimator). Random effects models were used based on the Bayesian theory framework. The node-splitting approach was generated to determine study network inconsistency. The convergence of the model was evaluated using the trajectory map, density map, and the potential scale reduction factor (PSRF). Rank sort graphs illustrate the best valve deployment techniques or valve types.

**Results:**

A total of 830 articles were searched referring to the incidence of CO using the valve deployment system of SEVs or BEVs during the TAVR procedure, from which 51 were included (27,784 patients). The procedure incidence of coronary obstruction was 0.4% for the SEVs and 0.6% for the BEVs. Treatment ranking based on network analysis revealed SAPIEN 3 (Edwards Lifesciences (Irvine, CA, USA)) possessed the best procedural CO incidence (0.05%) performance, whereas SAPIEN (Edwards Lifesciences (Irvine, CA, USA)) produced the worst (1.04%).

**Conclusions:**

Our study indicates that CO incidence was not reduced during TAVR with BEVs compared to SEVs. SAPIEN 3 and SAPIEN had the lowest and highest TAVR-associated CO rates, respectively. These findings suggest that the SAPIEN 3 valve may be the best choice for reducing CO risk, and future studies should focus on its applicability in different populations. More randomized controlled trials with head-to-head comparisons of SEVs and BEVs are needed to address this open question.

**The PROSPERO registration:**

CRD42024528269, https://www.crd.york.ac.uk/PROSPERO/view/CRD42024528269.

## 1. Introduction

Managing aortic valve disease has evolved beyond conventional surgical approaches, with the emergence of transcatheter aortic valve replacement (TAVR). Over two decades of rigorous clinical evaluation have established the safety and efficacy profile of TAVR, positioning it as the primary treatment option for high-risk patients with severe aortic stenosis [1, 2]. This minimally invasive technique offers excellent outcomes and clear advantages compared to open surgery, particularly in reducing procedural mortality and major complications [2, 3, 4, 5]. Following recent clinical research conducted worldwide, the indications for TAVR have been broadened, extending the patient population from those at high-risk to those at moderate [6, 7, 8] and even low-risk [3, 4, 9, 10, 11], for asymptomatic severe aortic stenosis [12], and with a trend towards younger patients [7, 13].

Coronary occlusion (CO) is a rare but potentially fatal complication of TAVR surgery. With an incidence rate of <1%, an occurrence of this complication presents a mortality rate of 40% to 50% [14]. Additionally, research has shown that patients with aortic valve stenosis undergoing TAVR have a high likelihood (proportion ranges from 40% to 75%) of suffering from coronary artery disease, regardless of their surgical risk classification [6, 7, 15]. Therefore, managing the coronary access is a crucial aspect of lifelong care for TAVR patients [16]. The most frequent mechanism of CO is the displacement of the calcified native valve leaflets over the coronary ostium [14, 17]. The primary risk factors for CO mainly include a valve-to-coronary ostium distance <4 mm, coronary artery height <10 mm, aortic sinus diameter <30 mm, severe valve calcification, female gender, advanced age, high valve implantation position, and valve-in-valve procedures (especially with stentless bioprosthetic or surgical valves) [14, 17, 18]. This condition is linked to anatomical and valve-related factors [14, 17, 18].

With the increasing popularity of TAVR and the expansion of indications to lower-risk and younger patient groups, there is a growing demand for the safety and effectiveness of TAVR devices. Since the inception of TAVR surgery, self-expanding valves (SEVs) and balloon-expandable valves (BEVs) have been the mainstay of clinical use [19, 20]. Without doubt, the differing constructions and release mechanisms of these two valves have the potential to exert varying influences on coronary artery occlusion. Comparative studies have been conducted to evaluate the outcomes of TAVR with different devices, and those before and after device updates, observing differences in complication rates [21, 22, 23, 24]. However, there is currently a lack of consensus on the relationship between device type and the occurrence of CO. In fact, there is limited direct comparison research on SEV and BEV devices, and a lack of extensive comparisons between different transcatheter heart valves (THVs) on the market, especially for comparing new generation prostheses.

Based on the above considerations, to summarize the impact of different TAVR devices on intraoperative CO incidence, we systematically evaluated the differences in CO incidence between SEVs and BEVs during TAVR and ranked specific valve types accordingly. This analysis aimed to provide meaningful guidance on the safety and effectiveness of TAVR devices.

## 2. Methods

### 2.1 Literature Search Strategy and Selection Criteria

This systematic review and meta-analysis were conducted and reported in adherence to current guidelines, including the Preferred Reporting Items for Systematic Reviews and Meta-Analyses (PRISMA), amendment to the Quality of Reporting of Meta-analyses statement (QUOROM), and recommendations from The Cochrane Collaboration and Meta-analysis Of Observational Studies in Epidemiology (MOOSE). This study has been registered in PROSPERO under the registration number CRD42024528269.

The primary outcome of interest was the incidence of CO during TAVR with BEVs and SEVs. To identify and include all relevant studies, electronic literature databases including PubMed, Embase and the Cochrane Library, were searched manually and automatically for relevant English articles using the following strategy: ((TAVR OR transcatheter aortic valve implantation OR transcatheter aortic valve replacement) OR (Balloon-expandable valve) OR (Self-expandable valve)) AND ((coronary obstruction) OR (CO)). Studies including randomized controlled trials, observational studies, and propensity pair-matched studies published in English between January 2009 and June 2023 were identified. Only studies reporting the CO incidence were included, while studies with a sample size of fewer than 15 patients were excluded. Studies were collected only if they reported an occurrence of intraoperative CO events during the procedure. Studies should provide specific details about the implantation mechanism (BEV, SEV, and SAVR) and the type of prosthesis (SAPIEN, CoreValve (Medtronic, Minneapolis, MN, USA), Others). According to the Valve Academic Research Consortium (VARC-1/2) criteria [25, 26], CO can be defined as angiographic or echocardiographic evidence of a new, partial or complete, obstruction of a coronary ostium, either by the valve prosthesis itself, the native leaflets, calcifications, or dissection, occurring during or after the TAVR procedure. Imaging studies (coronary angiography, intravascular ultrasound, multi-slice CT angiography, or echocardiography), surgical exploration, cardiac biomarker elevations, and ECG changes indicating new ischemia can provide corroborative evidence. Furthermore, cases of valve-in-valve transcatheter valve implantation (ViV-TAVR) or failed TAVR procedures that required conversion to surgery for other reasons were not included.

### 2.2 Data Extraction and Critical Appraisal

Selected articles underwent extensive evaluation at the title and abstract levels by two independent researchers (YFW and ZJW) to assess their potential inclusion in this meta-analysis. Following a full-text review, any duplicated data were deleted. Since the quality scoring in meta-analyses of observational studies is controversial, two researchers (YFW and ZQL) independently assessed each article using the Newcastle–Ottawa scale for observational studies. Discrepancies were resolved through third-party adjudication (YJZ).

### 2.3 Statistical Analyses

Meta and network meta-analysis were executed using statistical analysis software R-studio 4.3.1 (Posit Software, PBC, Boston, MA, USA) and R Packages (Comprehensive R Archive Network: https://cran.r-project.org/) “meta”, “netmeta”, “rjags”, “gemtc”, “codetools”, and “igraph”.

#### 2.3.1 Meta-Analysis for Single Rate on the CO Incidence

Firstly, a meta-analysis of the single rate of the incidence of CO was conducted in all the selected studies. The rate was combined using the metaprop function to evaluate the CO incidence. A normality test was performed on either the original rate or the sample rate calculated by the four estimating methods (“PRAW”, “PLN”, “PLOGIT”, “PAS”, “PFT”) [27, 28, 29]. The Freeman–Tukey double arcsine transformation (PFT), closest to the normal distribution, was finally chosen (W = 0.83983, *p* < 0.001). The forest plot was drawn, and the heterogeneity analysis was performed. Heterogeneity and inter-study variance were estimated by calculating Cochran’s Q, I^2^, and τ^2^ (Sidik–Jonkman estimator) values [30]. Specifically, an I^2^ value >50% was considered evidence of moderate or severe inconsistency. The sensitivity analysis was not performed because of the metaprop function characteristics in a one-arm study. A funnel plot was applied to assess potential publication bias [31].

#### 2.3.2 Meta-Analysis for BEVs and SEVs

Fixed effects models were used due to low heterogeneity in studies, and population and risk ratios (RRs) were calculated. Methods for estimating heterogeneity and inter-study variance have been described previously [30]. Subgroup analyses were conducted to explore potential sources of heterogeneity. Similar to the meta-analysis for the single rate, a funnel plot was utilized to assess publication bias across studies [31], and sensitivity analyses were performed to evaluate the robustness of the pooled results.

#### 2.3.3 Network Meta-Analysis for Different Mechanisms and Valves

Potential publication bias, heterogeneity, and among-study variance were assessed as previously mentioned. The potential for inconsistency is uncertain due to discrepancies between direct and indirect inferences in pairwise comparisons. Furthermore, the node-splitting approach was generated to determine study network inconsistency [32]. Consistency was noted if the node analysis produced a value of *p* > 0.05. The convergence of the model can then be evaluated after drawing the trajectory map and density map, and calculating the potential scale reduction factor (PSRF).

To compare different mechanisms of implantation (BEV, SEV, and SAVR) and types of valves, we carried out the indirect and mixed comparisons and reported odds ratios (ORs) using a random effects network meta-analysis based on a Bayesian framework. Forest plots were drawn to show the comparisons above. To find the superior treatment, we obtained the relative ranking probability for each mechanism or valve and the hierarchy of competing treatments by rank sorting, and rank sort graphs were performed. Individual and comprehensive sort results were calculated.

## 3. Results

### 3.1 Literature Search

**Fig. 1.**
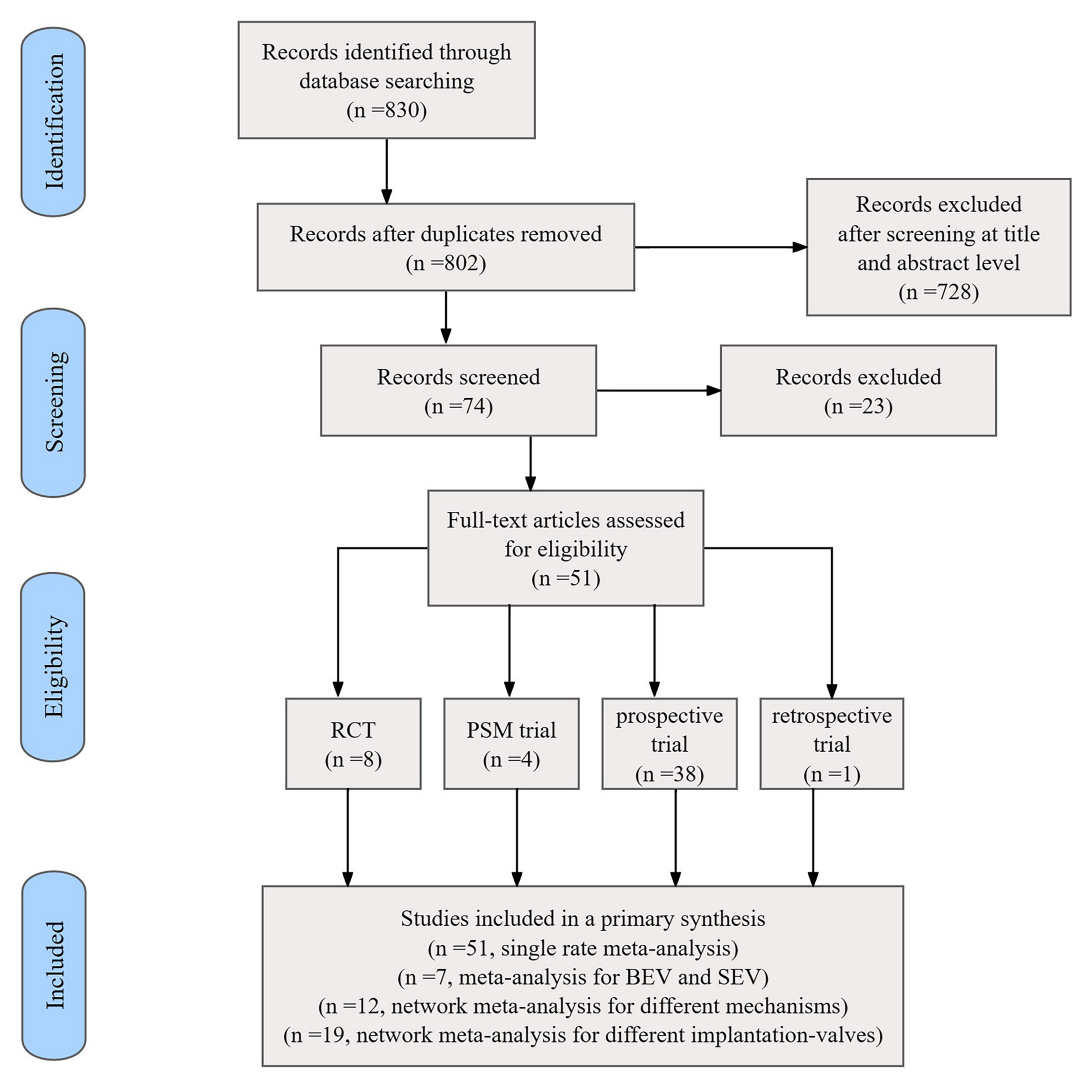
**Flowchart of the included studies**. RCT, randomized controlled trial; PSM, propensity score-matching; BEV, balloon-expandable valve; SEV, self-expandable valve.

A total of 830 citations were initially retrieved. Duplicates or irrelevant references reviewed according to title and abstract were excluded. Based on exclusions at the full-text level, 51 relevant articles (**Supplementary Table a**) were finally included after comparisons using pre-set criteria: randomized controlled trials (n = 8), propensity score-matched studies (n = 4), non-adjusted prospective studies (n = 38), and retrospective studies (n = 1). The selected studies reported on a total of 27,784 patients (10,749 patients receiving SEVs, 14,052 patients receiving BEVs, and 2983 patients receiving SAVR). Data were analyzed after unified formatting, and those presented as the median interquartile range instead of the mean ± standard deviation were transformed using online tools [33, 34, 35, 36], i.e., mean variance estimation (https://www.math.hkbu.edu.hk/~tongt/papers/median2mean.html). The operative risk was noted according to the operative risk models, the Society of Thoracic Surgeons score (STS score) [37]. If the STS score was not specified, the logistic EuroSCORE (EuroSCORE I) [38, 39] was used for risk classification. The quality assessments have been summarized in **Supplementary Tables 1–3**. The flowchart of the included studies can be found in Fig. 1.

### 3.2 Meta-Analysis for Single Rate on the CO Incidence

A total of 51 articles were analyzed. The patients and details for aortic valve implantation or study characteristics are presented in **Supplementary Table a**. The median study cohort size was 222 (inter quartile range, IQR 15–2688) patients. Approximately 45% (IQR 8.61%–80.0%) were male. Of the total cohort, 74.5% (38/51) of studies focused on patients with high operative risk according to the operative risk classification described above, seven studies focused on intermediate risk, and six on low risk. The CO incidence varied from 0% to 5.9%, while for procedures with BEVs, it varied from 0% to 5.9%, and SEVs from 0% to 1.0%. The CO incidence for the SAPIEN 3 valve was 0.05%, while the SAPIEN valve was 1.04% (**Supplementary Table b**). A meta-analysis showed that the rate of CO incidence during the TAVR procedure was 0.1479% but with high heterogeneity (I^2^ = 52%, τ^2^ = 0.0006, *p* < 0.01) (**Supplementary Fig. 1**). The publication bias was outstanding, and the trim-and-fill method was used (**Supplementary Fig. 2**).

### 3.3 Meta-Analysis for BEVs and SEVs

**Fig. 2.**
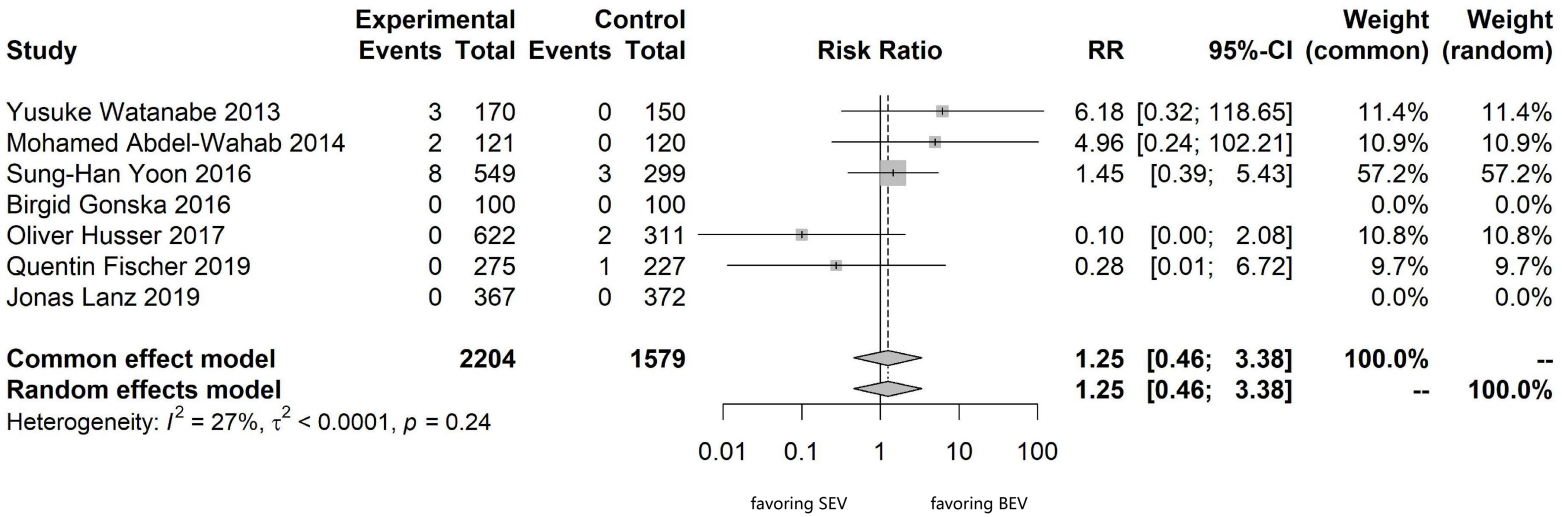
**Meta-analysis comparing SEVs and BEVs for the intraoperative CO incidence**. CO, coronary occlusion.

For this meta-analysis, seven studies comparing head-to-head BEVs and SEVs were included: randomized controlled trials (n = 2), propensity score-matched studies (n = 2), and non-adjusted prospective studies (n = 3). Pairwise meta-analysis comparing BEVs versus SEVs showed moderate heterogeneity (I^2^ = 27%, τ^2^ < 0.0001, *p* = 0.24) (Fig. 2). Subgroup analysis was performed but was unable to elucidate the origin of heterogeneity (**Supplementary document**; **Supplementary Fig. 3**). In the BEV group, the pooled incidence of CO was 0.6% (13 out of 2204), while in the SEV group, the incidence of CO was 0.4% (6 out of 1579). The RR of BEVs versus SEVs was 1.25 (95% CI: 0.46–3.38; *p* = 0.67). The funnel plot suggested an absence of significant bias in these studies, and the sensitivity analysis revealed the robustness of the meta-analysis findings (**Supplementary Figs. 4,5**). This analysis verified no noticeable difference in the probability of intraoperative CO occurrence between BEVs and SEVs. Given the small number of studies included in this analysis and the significant heterogeneity among studies within the subgroup, these pooled findings may not be persuasive. While our analysis confirms the multifactorial nature of CO post-TAVR, key technical variables such as implantation depth, angulation, and the temporal distribution of CO events are critical determinants in this complication. However, the limited availability of raw data precluded further stratification by these parameters. Thus, prospective trials incorporating standardized imaging and procedural data capture are warranted to elucidate these relationships further.

### 3.4 Network Meta-Analysis for Different Mechanisms and Implantation Valves

To address the comparison of mechanisms and implantation valves, 31 studies were considered for network meta-analysis by combining direct and indirect comparisons, including 12 studies comparing different mechanisms (BEVs, SEVs, and SAVR) and 19 studies comparing different implantation valves (SAPIEN, SAPIEN XT (Edwards Lifesciences, Irvine, CA, USA), SAPIEN 3, CoreValve, Evolut R (Medtronic, Minneapolis, MN, USA), Evolut PRO (Medtronic, Minneapolis, MN, USA), ACURATE neo (Boston Scientific, Marlborough, MA, USA), and SAVR valves).

#### 3.4.1 Network Meta-Analysis for Different Mechanisms

**Fig. 3.**
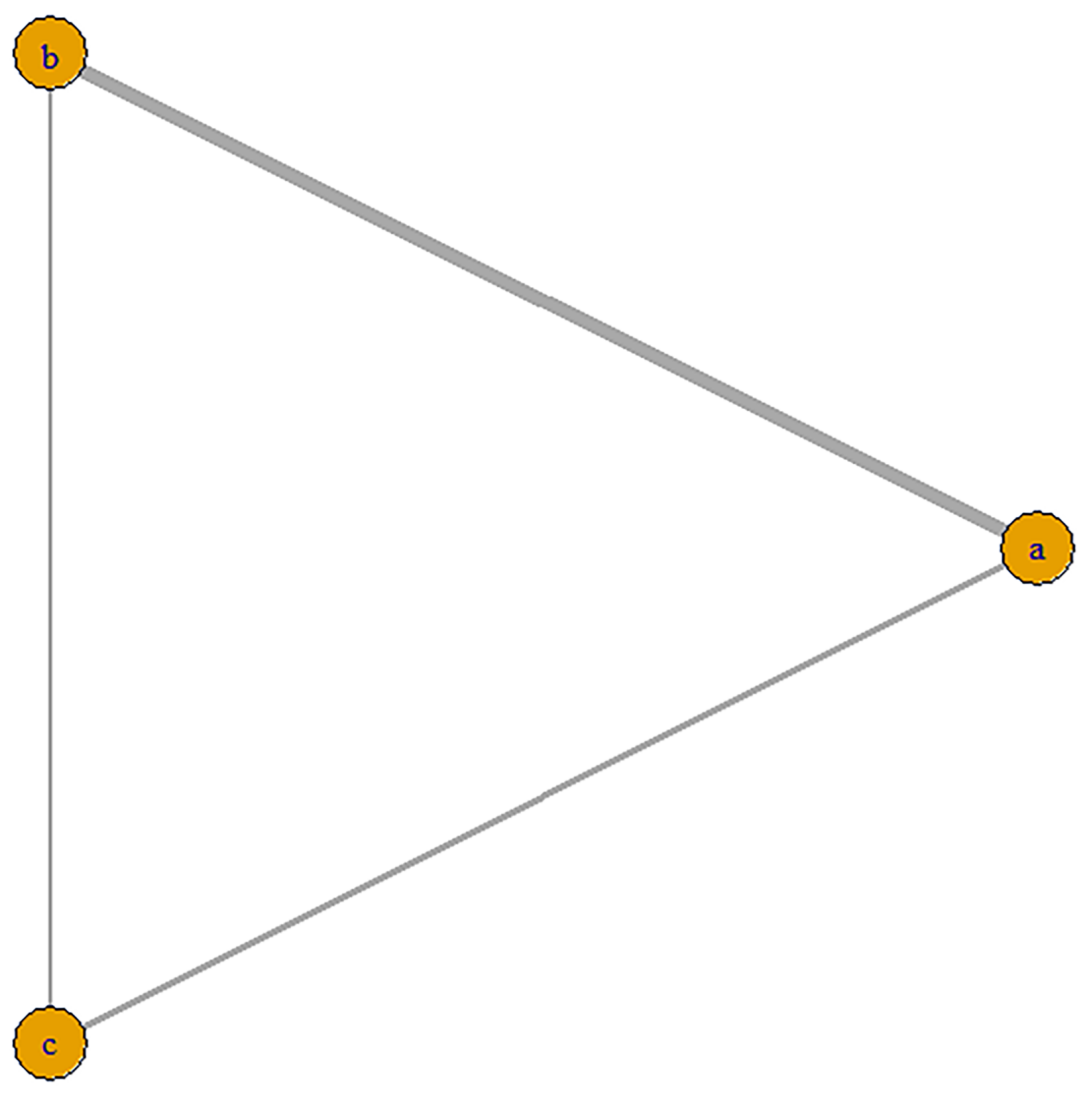
**Network diagram of the included studies based on the mechanisms of AVR implantation**. The thickness of the lines is directly proportional to the number of studies available for each direct comparison. a, BEVs; b, SEVs; c, SAVR. AVR, aortic valve replacement; SAVR, surgical aortic valve replacement.

**Fig. 4.**
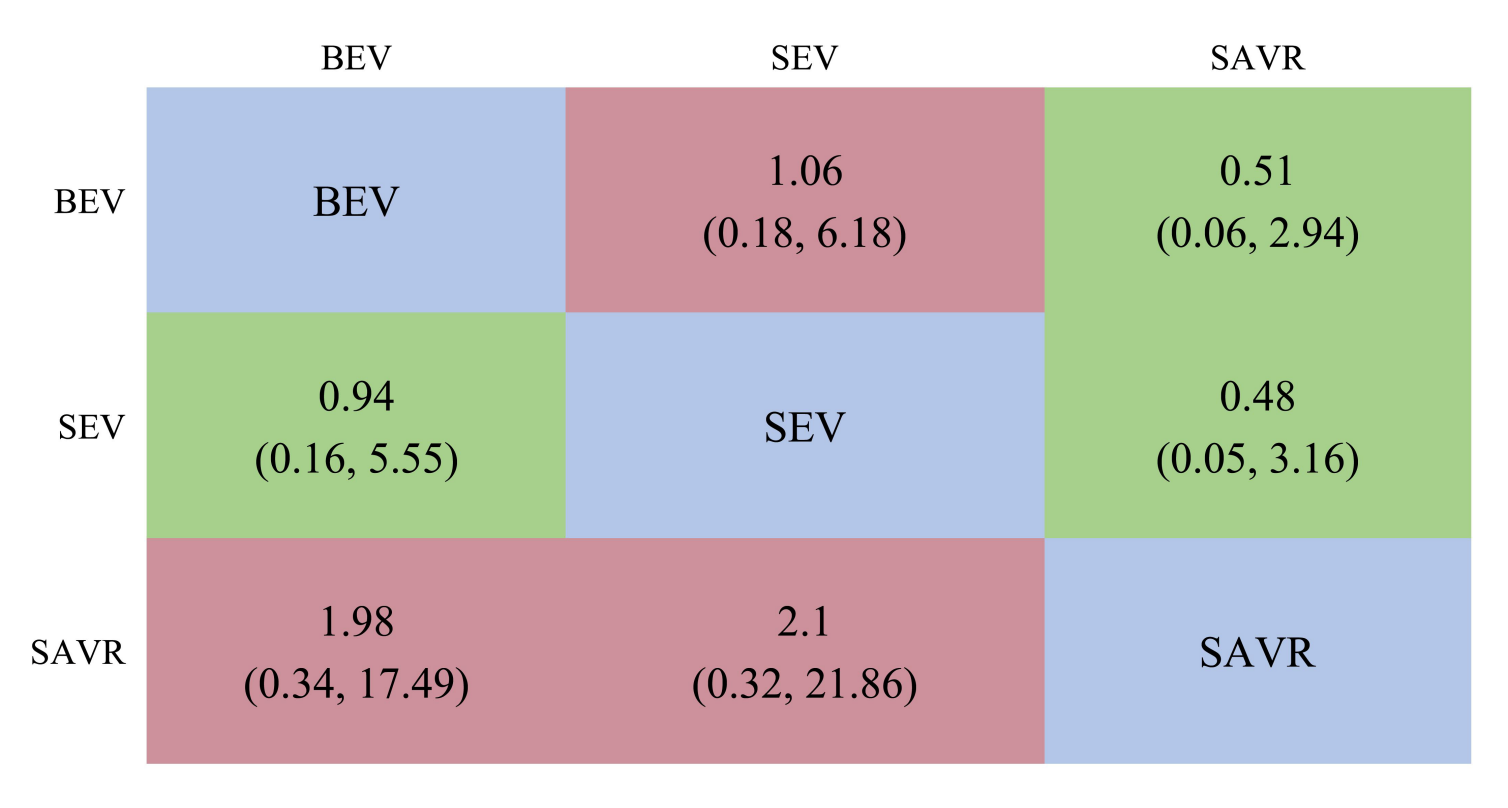
**A league table of the network comparison among the three implantation mechanisms: BEV, SEV, and SAVR**. Green blocks represent a positive effect; red blocks represent a negative effect; the diagonal elements of the league table,the blue squares, indicate comparisons between identical interventions.

**Fig. 5.**
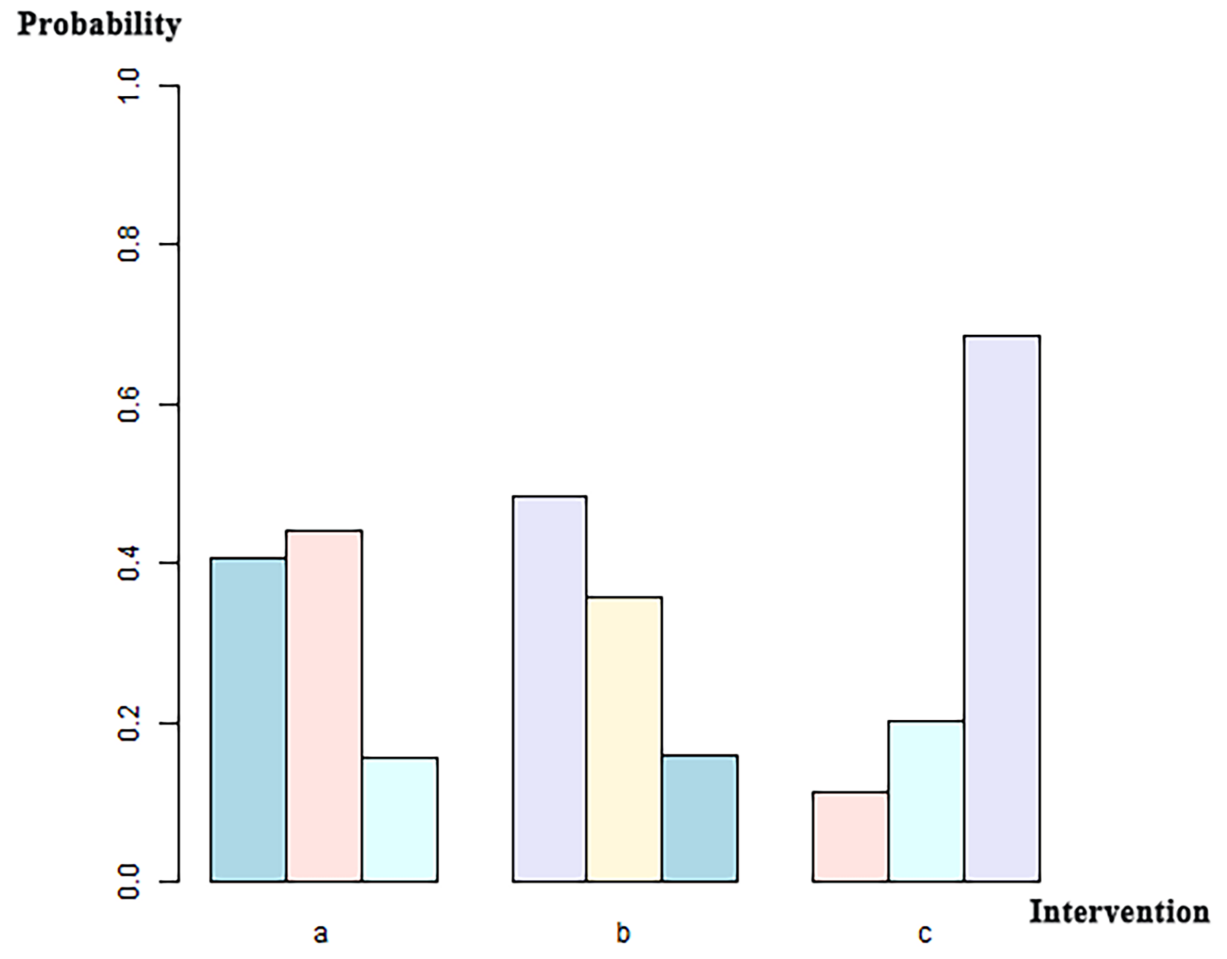
**Ranking sort chart for the three implantation mechanisms: BEV, SEV, and SAVR**. The x-axis labels correspond to distinct intervention groups (a, BEVs; b, SEVs; c, SAVR). For each intervention, the three bars (left to right in different colors) indicate the probability of ranking first, second, and third for CO incidence among all interventions, with probabilities quantified on the y-axis.

We included 12 studies, such as the PARTNER trial comparing SAVR with BEVs, the SURTAVR trial comparing SAVR with SEVs, and the CHOICE trial comparing SEVs with BEVs. Additionally, other relevant prospective clinical studies were included. In total, 9895 patients were included, of which 3745 received BEV-TAVR, 3167 received SEV-TAVR, and 2983 received SAVR. The network relationship diagram was drawn and provided (Fig. 3), and a consistency model was established. The league chart summarizes the network comparisons among the three interventions (Fig. 4): The SAVR group showed significantly lower CO risk compared to either the BEV (odds ratio, OR = 0.51) or SEV (OR = 0.48) groups. Patients receiving a BEV had a lower risk for CO than those receiving a SEV (OR = 0.94). However, the effects were not considered statistically significant. Based on the ranking sort chart (Fig. 5), the SAVR had the lowest occurrence of CO during aortic valve replacement, BEVs came in second, while SEVs performed the worst. The established consistency model exhibited good convergence (PSRF = 1.01) and satisfied the assumptions of consistency and homogeneity (**Supplementary Figs. 6–9**).

#### 3.4.2 Network Meta-Analysis for Different Implantation Valves

**Fig. 6.**
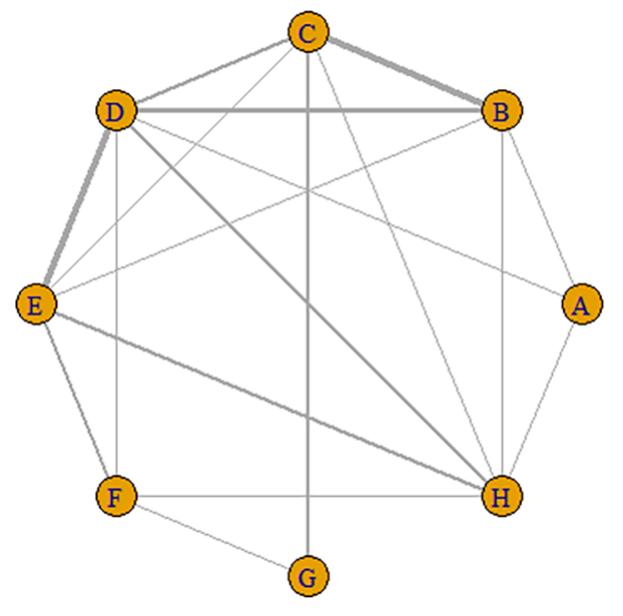
**Network diagram of the included studies based on the AVR implantation valves**. (1) Nodes: Each letter (A, B, C…) represents an intervention (A, SAPIEN; B, SAPIEN XT; C, SAPIEN 3; D, CoreValve; E, CoreValve Evolut R; F, CoreValve Evolut PRO; G, ACURATE neo; H, SAVR valves). (2) Edges: line width corresponds to the number of direct clinical trial comparisons.

**Fig. 7.**
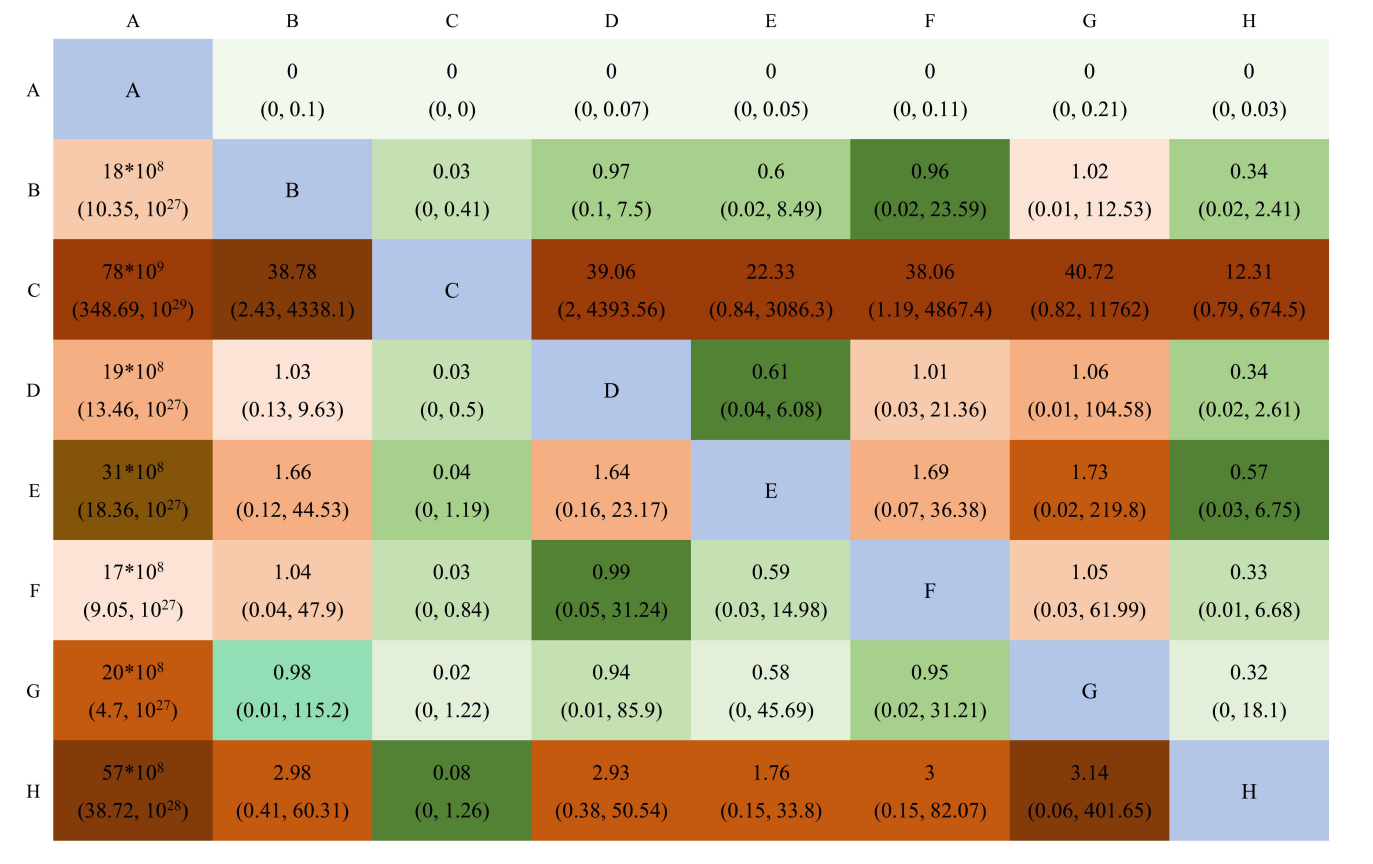
**A league table of the network comparison among the eight implantation valves: A, SAPIEN; B, SAPIEN XT; C, SAPIEN 3; D, CoreValve; E, CoreValve Evolut R; F, CoreValve Evolut PRO; G, ACURATE neo; and H, SAVR**. Green blocks depict a significantly higher CO incidence than the control; red blocks represent a significantly lower CO incidence than the control. Lighter colors indicate smaller effect sizes. The diagonal elements of the league table,the blue squares, indicate comparisons between identical interventions.

**Fig. 8.**
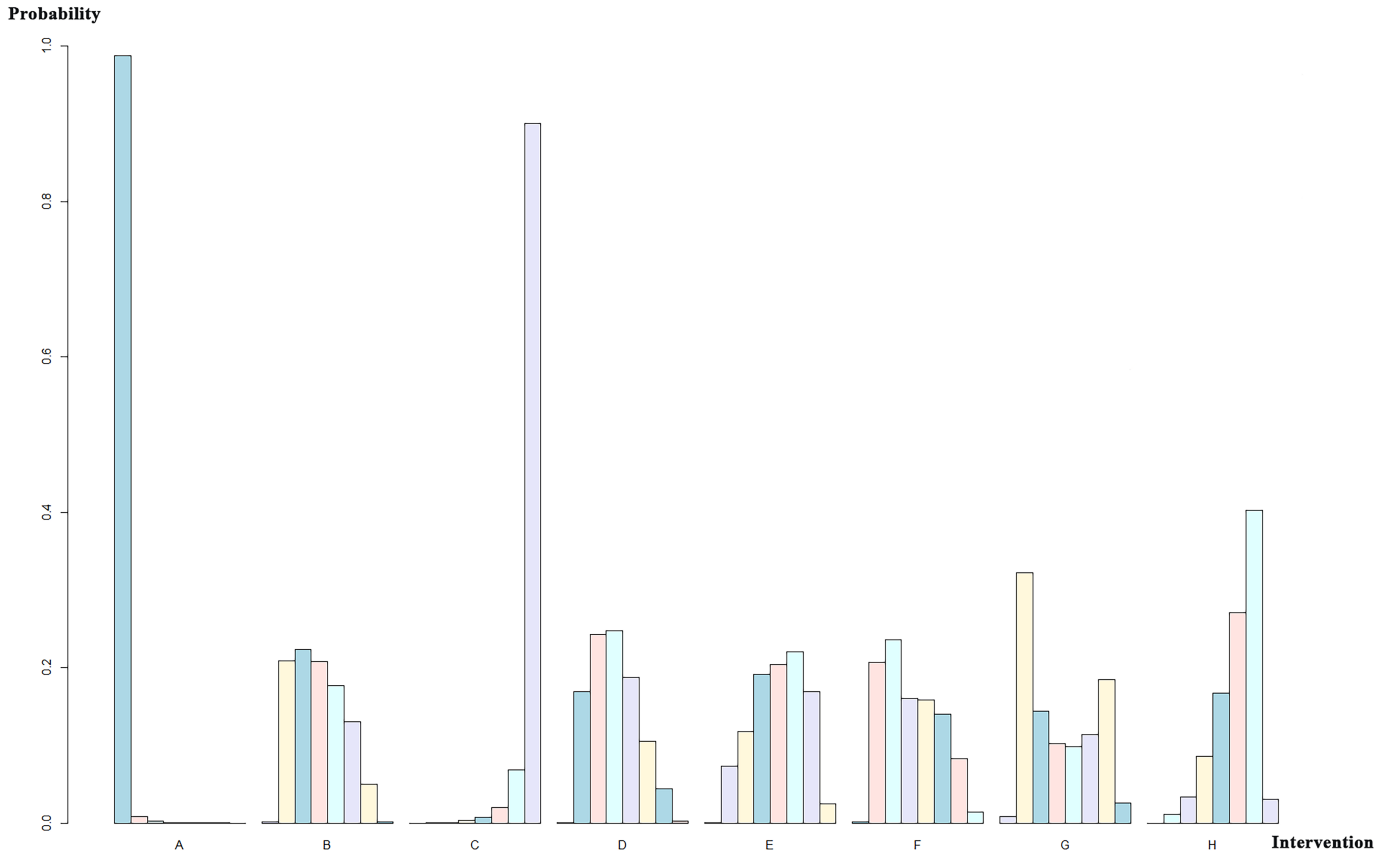
**Ranking sort chart for different valves used in AVR implantation**. The x-axis labels correspond to distinct intervention groups (A, SAPIEN; B, SAPIEN XT; C, SAPIEN 3; D, CoreValve; E, CoreValve Evolut R; F, CoreValve Evolut PRO; G, ACURATE neo; H, SAVR valves). For each intervention, the bars (left to right in different colors) indicate the probability of being ranked 1st to 8th for CO incidence among all interventions, with probabilities quantified on the y-axis.

A total of 19 studies with 12,904 patients were included and were divided into eight groups: SAPIEN, SAPIEN XT, SAPIEN 3, CoreValve, Evolut R, Evolut PRO, ACURATE neo, and SAVR valves. The network relationship diagram was drawn and is provided (Fig. 6). The pooled CO rates for each group are shown in **Supplementary Table b**. The league chart summarizes the network comparisons among the groups (Fig. 7). SAPIEN 3 showed the lowest CO risk compared to the others. Conversely, SAPIEN showed the highest CO risk. The difference was statistically significant. Based on the ranking chart (Fig. 8), SAPIEN 3 presents the lowest occurrence of CO during aortic valve replacement. Other groups had no significant difference and came in second together, while SAPIEN performed the worst.

Furthermore, the model exhibited acceptable convergence (PSRF = 1.1) and adhered to the assumptions of consistency and homogeneity (**Supplementary Figs. 10–13**).

## 4. Discussion

CO is one of the most dangerous complications associated with TAVR; despite its relatively low occurrence rate, it carries a significant risk of mortality [14]. The most frequent mechanism is the displacement of the calcified native valve leaflets over the coronary ostium [14, 17]. The clinical presentation of CO includes persistent severe hypotension, ST-segment changes, and ventricular arrhythmias. These complications usually occur immediately following valve implantation [40, 41]. The left main artery is the most frequently involved, accounting for over 70% of cases, while right artery occlusion is rare [41]. In terms of preventing CO in high-risk patients, consideration can be given to protecting the coronary arteries with a guidewire and an unexpanded balloon or stent. The stent can be pulled back and deployed in a “chimney” configuration to maintain coronary patency [42, 43]. However, it is worth noting that the risk cannot always be eliminated. Currently, patients with medium or low surgical risk and younger age groups can be evaluated for TAVR. As for patients with asymptomatic severe AS in gray areas where guidelines do not provide clear assistance, the AVATAR trial showed that the prognosis of patients who do not receive intervention is poor [44]. TAVR potentially expands the benefits to patients not previously candidates and sparks interest in early intervention. Therefore, more comprehensive comparisons and evaluations of TAVR complications are crucial. TAVR-related CO remains a significant cause of mortality and morbidity. While the mainstream TAVR valve systems—SEVs and BEVs—are widely utilized, limited research exists exploring differences in the incidence of CO between these valve systems. Furthermore, research should focus on valve types to provide practical guidance for clinical decision-making.

This article reviewed CO events during TAVR in around 27,784 patients across the literature. The key findings can be summarized as follows. (a) The current rate of TAVR-related CO occurrences was 0.15% (when conducting a single-rate analysis of CO events, we excluded articles that solely utilized SAVR procedures). For articles that compared SAVR and TAVR, we only extracted and analyzed information from patients who underwent TAVR procedures. (b) There was no noticeable difference in the probability of intraoperative CO occurrence between BEVs (0.6%) and SEVs (0.4%). (c) SAVR groups showed lower CO risk than the BEV (OR = 0.51) or SEV (OR = 0.48) groups. (d) SAPIEN 3 demonstrated the lowest incidence of CO, whereas the first-generation SAPIEN exhibited the highest CO occurrence. The mean CO rate was 1.04% for the SAPIEN valve and 0.05% for the SAPIEN 3 valve.

### 4.1 BEVs, SEVs, and Their Different Valves

According to previous clinical trials examining valve systems, with limited extension of the valve frame beneath the aortic valve ring, a BEV minimizes instrumental manipulation within calcified and degenerated valves. Further, BEVs offer convenient implantation and a lower post-valve replacement (PVR) incidence. However, most previous studies reported higher CO incidences during TAVR were associated with BEVs [41, 45, 46]. Previous registry data also indicated a slightly higher occurrence of CO following BEV implantation (<0.4%) compared to SEV implantation (<0.2%) [47, 48, 49, 50]. For example, a study compared the SAPIEN and the CoreValve; notably, the CO rate in the BEV group was higher than the SEV group (1.8% vs. 0%) [51]. Moreover, the CHOICE randomized controlled trial [52] compared the performance of the SAPIEN XT with that of the CoreValve and formulated the same conclusion; likewise, the Asian TAVR study [53].

However, our meta-analysis demonstrated non-inferiority of BEVs to SEVs in CO risk, with no significant between-group differences observed. The network meta-analysis further identified the SAPIEN 3 system as having the lowest CO incidence, contrasting with the highest rates seen in its predecessor, SAPIEN, thereby positioning the SAPIEN 3 system as the safest valve for this safety endpoint.

To explain the study conclusion, we first delve into the strengths of SEVs: (1) The BEV and SEV (SAPIEN and CoreValve) differ in requirements for the minimum aortic sinus diameter and coronary ostium height. The manufacturer offers recommendations for implanting the CoreValve system, specifying an aortic sinus width ≥27 mm for the 26 mm model and ≥28 mm for the 29 mm model, along with a coronary ostium height of ≥14 mm. While not all centers strictly adhere to these recommendations, these guidelines may have prevented numerous CO events in the CoreValve system. (2) Subsequently, following technological advancements, commissural alignment techniques have been applied to SEVs, significantly reducing the occurrence of CO. With their advanced commissural alignment technology, the Evolute PRO and ACURATE neo2 (Boston Scientific, Marlborough, MA, USA) valves effectively address the issue of the commissural junction in front of the coronary ostium. (3) Repositioning SEVs is also often possible [54]. For instance, the J-VALVE (JieCheng Medical Technology Co., Ltd., Suzhou, Jiangsu, China) SEV has a positioning key and a clamping effect between the valve and the valve leaflets, which can avoid blocking the coronary artery. Nonetheless, there is a lack of comprehensive research on BEVs and SEVs, and the present findings have significant limitations.

Advantages of SEVs primarily stem from updated manufacturing techniques and operations, meaning a thorough comparison with the updated BEVs is necessary to provide a comprehensive assessment. In a study examining a new generation of BEVs, SAPIEN 3 and CoreValve had the same CO rate [55]. The SCOPE I [24] trial compared SAPIEN 3 with the ACURATE neo, both of which had a CO rate of 0%. These two studies demonstrate that there is no difference in the occurrence of CO between BEVs and SEVs. Additionally, some studies have revealed that the SEV may be associated with a higher incidence of intraoperative CO [17, 23, 56].

In our meta-analysis, SAPIEN 3 demonstrated the lowest CO rates. We further elucidate how the SAPIEN 3 device reduces CO. (1) Enhanced implantation precision and anatomical compatibility: The SAPIEN 3 system incorporates radiopaque markers and a stepwise deployment mechanism via the commander delivery system, enabling precise positioning of the valve frame relative to the aortic annulus. This reduces excessive protrusion into the left ventricular outflow tract (LVOT), a critical factor in CO risk. In contrast, SEV systems (e.g., CoreValve Evolut PRO) rely on passive self-expansion, which may lead to unpredictable frame migration [3, 57, 58, 59, 60, 61]. The lower-profile 14-French sheath of SAPIEN 3 (vs. 18-French in SAPIEN XT) minimizes vascular trauma and improves maneuverability in tortuous anatomies, reducing malposition-related CO risks [57, 62]. (2) Optimized frame geometry and radial forces. The SAPIEN 3 frame (18.7–22.5 mm) is shorter than its predecessor (SAPIEN XT: 22.5–27.5 mm) and significantly shorter than SEV frames (Evolut PRO: 52 mm) [63, 64]. This design limits mechanical interaction with the LVOT and coronary ostia [65], particularly in patients with horizontal aortic roots or low-lying coronary arteries [66, 67, 68]. BEVs achieve immediate and predictable radial force upon balloon expansion, avoiding the progressive expansion seen in SEVs (e.g., ACURATE neo). This stability reduces late-onset CO caused by delayed frame expansion [69]. (3) Coronary access preservation. The open-cell architecture of SAPIEN 3 [57] at the inflow portion (vs. the closed-cell distal design in Evolut PRO) facilitates potential future coronary access [63, 64]. The 3.5–4.0 mm strut-free zones in SAPIEN 3 also allow easier catheter passage than SEV systems [57, 63]. Moreover, the alignment markers of SAPIEN 3 assist in orienting the valve to avoid coronary ostia overlap [63]. In contrast, the supra-annular SEV designs (e.g., Evolut PRO) may displace native leaflets toward the coronary ostia, increasing CO risk. (4) Procedural standardization. BEV deployment via rapid pacing and balloon inflation standardizes the procedure, reducing operator-dependent variability in deployment depth—a key CO risk factor. The SURTAVI trial highlighted that SEV systems required more frequent repositioning, increasing malposition risks [6]. Conversely, the compatibility of SAPIEN 3 with CT-based predictive software (e.g., HeartNavigator, Edwards Lifesciences) enables preprocedural planning to assess coronary ostia height and sinus of Valsalva dimensions, further mitigating CO risk [70, 71, 72, 73].

Based on the above analysis, both valve systems can achieve similar rates of CO through technical improvements, indicating no noticeable difference in the probability of intraoperative CO occurrence between BEVs and SEVs. The SAPIEN 3 valve should be the preferred choice for patients at high risk of CO. Nonetheless, large-scale randomized controlled trials are needed to improve validation of the long-term impact of valve types on CO risk.

### 4.2 SAVR, BEVs and SEVs

Finally, patients with low coronary ostia, severe calcification, and anatomical structures unsuitable for TAVR or high risk of post-TAVR paravalvular leakage may undergo SAVR. Moreover, SAVR has advantages in terms of CO because the commissural posts and commissure of the autologous aortic valve are aligned and away from the coronary ostia during SAVR, which TAVR cannot achieve. Currently, no available device or technology in the TAVR market can consistently achieve commissural alignment. The commissural posts of THVs create physical obstacles when facing the coronary ostia, making the coronary access (CA) more challenging.

After reviewing the data collected in this study, our conclusion is consistent with previous perspectives: in the STACCATO trial [56], the BEV-TAVR had a higher CO rate than SAVR. Similarly, in the Evolut Low Risk Trial [4], the SEV-TAVR had a higher CO rate than SAVR. In summary, SAVR is generally believed to prevent CO, but it is not without the possibility of CO occurrence [74, 75]. Our network meta-analysis demonstrates that the SAVR group exhibits significantly lower CO risk than the BEV (OR = 0.51) and SEV (OR = 0.48) groups. However, the league table suggests a lack of statistical significance, possibly due to the small number of included studies and numerous confounding factors. In addition, we made an interesting finding in our research: The CO rate for TAVR (SAPIEN XT) was 0.4% (4/1011) in the PARTNER II cohort A trial [7], while for SAVR it was 0.6% (6/1021). In the PARTNER III trial [3, 76], the CO rate for the TAVR (SAPIEN 3) was 0.2% (1/496), while for SAVR it was 0.4% (2/454). This could also be one of the reasons for the lack of statistical significance. Furthermore, considering the results of the valve-related CO incidence in this article, the SAPIEN 3 valve is associated with the lowest occurrence of CO, which may provide further evidence for the advantages of the SAPIEN 3 valve. Of course, the current results are insufficient to conclude the non-inferiority of TAVR in CO incidence; however, we do not believe a CO occurrence difference exists among SAVR, BEVs, and SEVs. Nevertheless, some considerations may be made about the safety of TAVR procedures, valve system selection, and the broader application of TAVR. As TAVR technology and learning curves continue to develop, randomized controlled trials comparing newer and improved SEVs and BEVs are needed to address this unresolved issue directly.

## 5. Limitations

This investigation presents several limitations. Many articles fail to pay attention to the documentation of CO events, resulting in a limited number of studies suitable for analysis, and the heterogeneity among studies is not low. Although the present network meta-analysis was based on published studies, publication bias remains a weakness. Moreover, articles focusing on comparisons between new generation valves are currently lacking. Studies with small sample sizes were not included in the final analysis, potentially leading to information loss. This analysis focused on study-level data, but exploring individual patient data could offer additional insights. In addition, the occurrence of CO following TAVR is multifactorial and influenced by critical procedural factors such as valve implantation depth and angulation. Furthermore, time distributions of CO events (e.g., immediate post-procedure vs. delayed onset within hours) and their association with time-incidence curves hold significant clinical relevance. However, limitations persist due to the limited number of available studies and insufficient raw datasets. Specifically, detailed procedural metrics (e.g., quantitative angulation data) and temporal event distributions were not consistently reported across included trials, precluding comprehensive stratified analyses of these factors.

## 6. Conclusions

The rate of TAVR-associated CO is similar between BEVs and SEVs. This network meta-analysis identified SAPIEN 3 and SAPIEN as the valves with the lowest and highest TAVR-associated CO rates, respectively.

Device-based considerations should be conducted when performing patient selection and informed consent regarding information and prediction of TAVR-associated CO, particularly in the light of an extension of patient selection (younger and lower-risk patients) for TAVR. This study provides systematic evidence for valve selection in TAVR, aiding in optimizing clinical decision-making.

## Data Availability

All data reported in this paper can be available from the corresponding author on reasonable request.

## Abbreviations

BEV, balloon-expandable valves; CO, coronary obstruction; LVOT, left ventricular outflow tract; OR, odds ratio; PRISMA, Preferred Reporting Items for Systematic Reviews and Meta-Analyses; PSRF, Potential Scale Reduction Factor; QUOROM, Quality of Reporting of Meta-analyses; RR, risk ratio; SEV, self-expanding valves; SAVR, surgical aortic valve replacement; STS score, the society thoracic of surgeons score; TAVR, transcatheter aortic valve replacement; THVs, transcatheter heart valves; VARC, valve academic research consortium.

## Supplementary Material

Supplementary material associated with this article can be found, in the online version, at https://doi.org/10.31083/RCM36208.
