# Supplementary material for: Incidence of Coronary Obstruction During Aortic Valve Implantation: Meta-Analysis and Mixt-Treatment Comparison of Self-Expandable Versus Balloon-Expandable Valve Prostheses
Source: Rev Cardiovasc Med. 2025 Jul 29;26(7):36208. doi: 10.31083/RCM36208 (PMC12326413; doi:10.31083/RCM36208)
Supplement: Supplementary file 1 [file 2153-8174-26-7-36208-s1.zip › Supplementary document.docx]

**1.*Meta-analysis for BEV and SEV***

To provide a nuanced understanding of the findings, subgroup analysis and publication bias testing were further conducted. Subgroup analysis was performed taking into account multiple dimensions, such as the research types, the risk stratification of the patient enrolled, and the variations in TAVR approach types (Figure S3). However, the results were unable to elucidate the origin of heterogeneity, and each subgroup consistently demonstrated a lack of distinguishable differences between BEV and SEV. The Funnel plot, which was created to investigate the potential for publication bias, suggested an absence of significant bias in these studies (Figure S4).

The sensitivity analysis revealed that even after excluding individual studies one at a time, the pooled results consistently demonstrated no significant difference between BEV and SEV, indicating the robustness of the meta-analysis findings (Figure S5). Therefore, this analysis verified that there is no noticeable difference in the probability of intraoperative CO occurrence between BEV and SEV.

***2. Network meta-analysis for different mechanisms and valves***

***2.1. Network meta-analysis for different mechanisms***

We evaluated the convergence of the model. As the number of iterations exceeded 5000, the MCMC chain displayed stable fluctuations with good overlap, evident in the trajectory plot. The density plot showed that as the number of iterations reached 20,000, the Bandwidth approached 0 and stabilized, indicating good convergence of the model (Figure S6). Moreover, the Potential Scale Reduction Factor (PSRF) was calculated to be close to 1 (Figure S7), which, along with the other findings, strongly suggested that this model has achieved satisfactory convergence.

Additionally, it is essential to verify the heterogeneity and consistency of the model. According to the nodal analysis diagram (Figure S8), the direct comparison, indirect comparison and network comparison results among interventions SEV-TAVR, BEV-TAVR and SAVR, had P-values exceeding 0.05, suggesting a lack of significant statistical difference and satisfactory consistency.

As shown in Figure S9, the direct comparison between interventions SEV-TAVR and BEV-TAVR exhibited an I^2^ of 22.2%, while the network comparison result had an I^2^ of 20.5%. Similarly, the direct comparison between interventions SAVR and BEV-TAVR displayed an I^2^ of 38.5%, with the network comparison result showing an I^2^ of 52.2%. In addition, the direct comparison between interventions SAVR and SEV-TAVR had an I^2^ of 59.5%, while the network comparison result had an I^2^ of 3.5%. Therefore, they generally met the assumption of homogeneity. Overall, the established consistency model fitted well and satisfied the assumptions of consistency and homogeneity, providing support for the reliability and validity of our analysis.

***3.2. Network meta-analysis for different valves***

We evaluated the convergence of the model. Methods were the same with **Section 2.1**, and the PSRF was calculated to be 1.1 (Figure S11). Taking findings about the MCMC chain and the Bandwidth into consideration (Figure S10), this model had acceptable convergence.

Similarly, the methods for assessing model consistency and heterogeneity followed the description provided in Section **2.1**, and the results were presented in Figure S12-13. Upon examination of the provided figure, it was evident that the consistency model previously adhered to the assumptions of consistency and homogeneity.
