# Supplementary material for: Incidence of Coronary Obstruction During Aortic Valve Implantation: Meta-Analysis and Mixt-Treatment Comparison of Self-Expandable Versus Balloon-Expandable Valve Prostheses
Source: Rev Cardiovasc Med. 2025 Jul 29;26(7):36208. doi: 10.31083/RCM36208 (PMC12326413; doi:10.31083/RCM36208)

**Supplementary Table 1. JBI for the quality assessment of the risk of bias in studies analyzed for single-rate**

| AUTHOR | 1 | 2 | 3 | 4 | 5 | 6 | 7 | 8 | 9 | 10 |
| --- | --- | --- | --- | --- | --- | --- | --- | --- | --- | --- |
| Josep Rodés-Cabau | Y | N | Y | Y | Y | Y | N | Y | Y | Y |
| Thomas | Y | Y | Y | Y | Y | Y | Y | Y | Y | Y |
| Walther 2011 | Y | N | Y | Y | Y | Y | N | Y | Y | Y |
| Walther 2012 | Y | N | Y | Y | Y | Y | Y | Y | Y | Y |
| Treede | Y | N | Y | Y | Y | Y | Y | Y | N | Y |
| J. Muñoz-García | Y | Y | Y | Y | Y | Y | Y | Y | Y | Y |
| Webb | Y | Y | Y | Y | Y | Y | Y | Y | Y | Y |
| Maeda | Y | Y | Y | Y | Y | Y | Y | Y | N | Y |
| Manoharan | Y | Y | Y | Y | Y | Y | Y | Y | N | Y |
| Schymik | Y | Y | Y | Y | Y | Y | Y | Y | Y | Y |
| G.P.Ussia | Y | Y | Y | Y | Y | Y | Y | Y | Y | Y |
| Linke | Y | Y | Y | Y | Y | Y | Y | Y | Y | Y |
| Bapat | Y | Y | Y | Y | Y | Y | N | Y | Y | Y |
| Vahanian | Y | Y | Y | Y | Y | Y | Y | Y | Y | Y |
| Kodali | Y | Y | Y | Y | Y | Y | Y | Y | Y | Y |
| Silaschi | Y | Y | Y | Y | Y | Y | Y | Y | Y | Y |
| Jochen Wöhrle | Y | Y | Y | Y | Y | Y | Y | Y | Y | Y |
| Miura | Y | Y | Y | Y | Y | Y | Y | Y | Y | Y |
| J. Popma | Y | Y | Y | Y | Y | Y | Y | Y | Y | Y |
| Wendler | Y | Y | Y | Y | N | Y | Y | Y | Y | Y |
| Silaschi | Y | Y | Y | Y | Y | Y | Y | Y | N | Y |
| Grube | Y | Y | Y | Y | N | Y | Y | Y | Y | Y |
| Khaled Al-Shaibi | Y | N | Y | Y | Y | Y | N | Y | N | Y |
| Garrido | Y | N | Y | Y | Y | Y | N | Y | Y | Y |
| Rodés-Cabau | Y | Y | Y | Y | Y | Y | Y | Y | N | Y |
| Tchétché | Y | Y | Y | Y | Y | Y | N | Y | Y | Y |
| Won-Keun Kim | Y | Y | Y | N | Y | Y | Y | Y | Y | Y |
| Manoharan | Y | Y | Y | Y | Y | Y | Y | Y | Y | Y |
| Saia | Y | Y | Y | Y | Y | Y | Y | Y | Y | Y |
| Ulrich Schäfer | Y | Y | Y | Y | Y | Y | Y | Y | N | Y |
| Yong | Y | Y | Y | Y | Y | Y | Y | Y | Y | Y |
| John K. Forrest | Y | Y | Y | Y | N | Y | N | Y | Y | Y |

Note: (l) Were there clear criteria for inclusion in the case series?; (2) Was the condition measured in a standard, reliable way for all participants included in the case series?; (3) Were valid methods used for identification of the condition for all participants included in the case series?; (4) Did the case series have consecutive inclusion of participants?; (5) Did the case series have complete inclusion of participants?; (6) Was there clear reporting of the demographics of the participants in the study?; (7) Was there clear reporting of clinical information of the participants?; (8) Were the outcomes or follow up results of cases clearly reported?; (9) Was there clear reporting of the presenting site(s)/clinics(s) demographic information?; (10)Was statistical analysis appropriate? Y: Yes; N: No.

**Supplementary Table 2-1.**  **Meta-analysis for BEV and SEV: Newcastle-Ottawa Scale for the quality assessment of the risk of bias in individual non-randomized studies**

| AUTHOR | SCORE | SELECTION | COMPARABILITY | OUTCOME/EXPOSURE |
| --- | --- | --- | --- | --- |
| Watanabe | 9 | **** | ** | *** |
| Sung | 9 | **** | ** | *** |
| Gonska | 9 | **** | ** | *** |
| Husser | 9 | **** | ** | *** |
| Fischer | 8 | **** | * | *** |

**Supplementary Table 2-2. Meta-analysis for BEV and SEV: Cochrane for the quality assessment of the risk of bias in RCT studies**


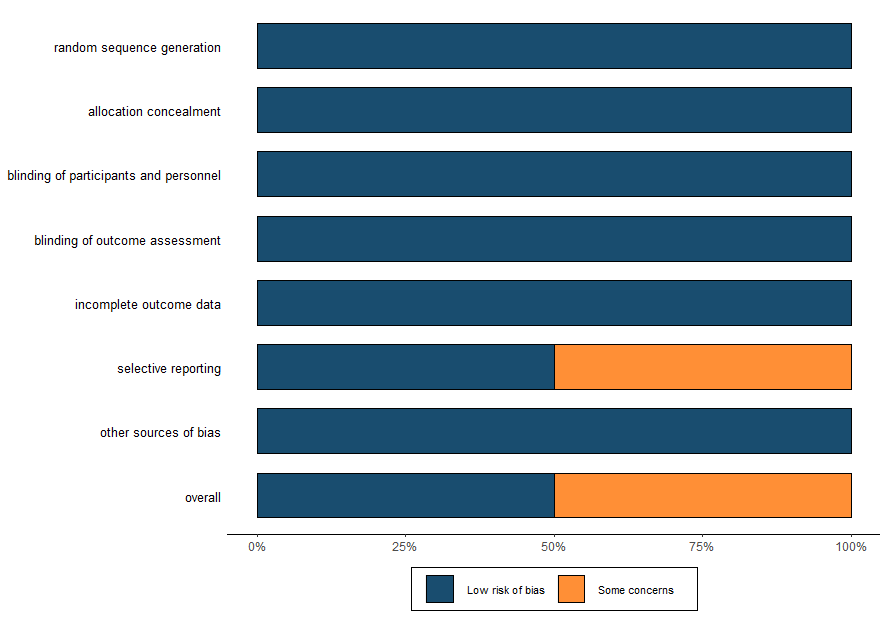

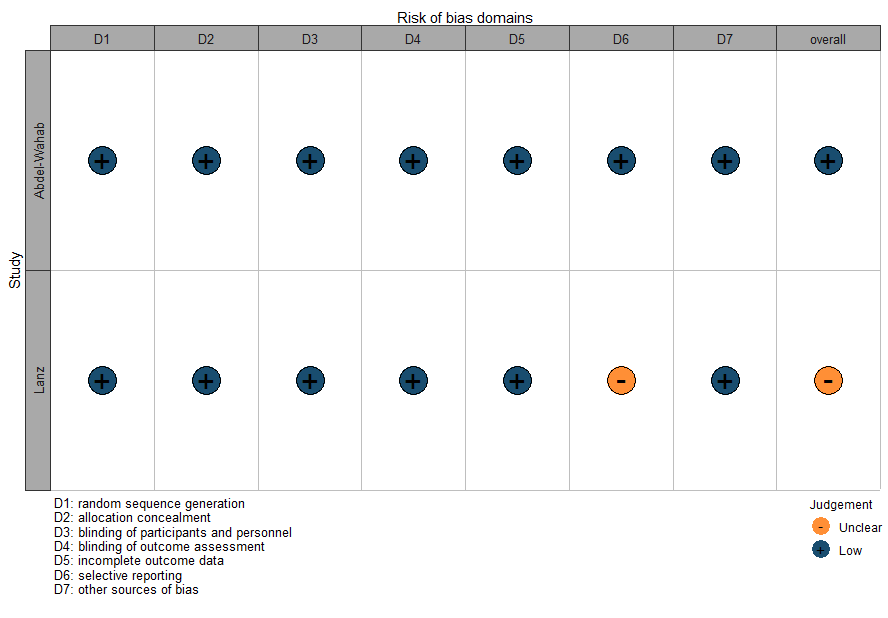


**Supplementary Table 3-1. Network meta-analysis for different mechanisms and valves: Newcastle-Ottawa Scale for the quality assessment of the risk of bias in individual non-randomized studies**

| AUTHOR | SCORE | SELECTION | COMPARABILITY | OUTCOME/EXPOSURE |
| --- | --- | --- | --- | --- |
| Watanabe | 9 | **** | ** | *** |
| Seiffert | 9 | **** | ** | *** |
| Bocksch | 7 | **** | ** | * |
| Petzina | 8 | **** | ** | ** |
| Sung | 9 | **** | ** | *** |
| Gonska | 9 | **** | ** | *** |
| Husser | 9 | **** | ** | *** |
| Nakashima | 8 | **** | * | *** |
| Hellhammer | 7 | **** | * | ** |
| Fischer | 8 | **** | * | *** |
| Pagnesi | 8 | **** | ** | ** |

**Supplementary Table 3-2. Network meta-analysis for different mechanisms and valves: Cochrane for the quality assessment of the risk of bias in RCT studies**


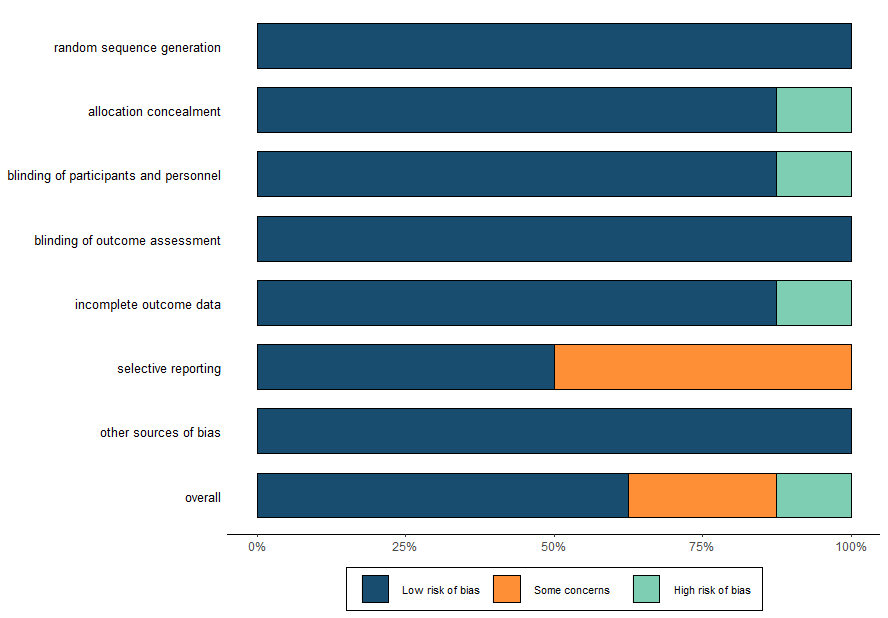

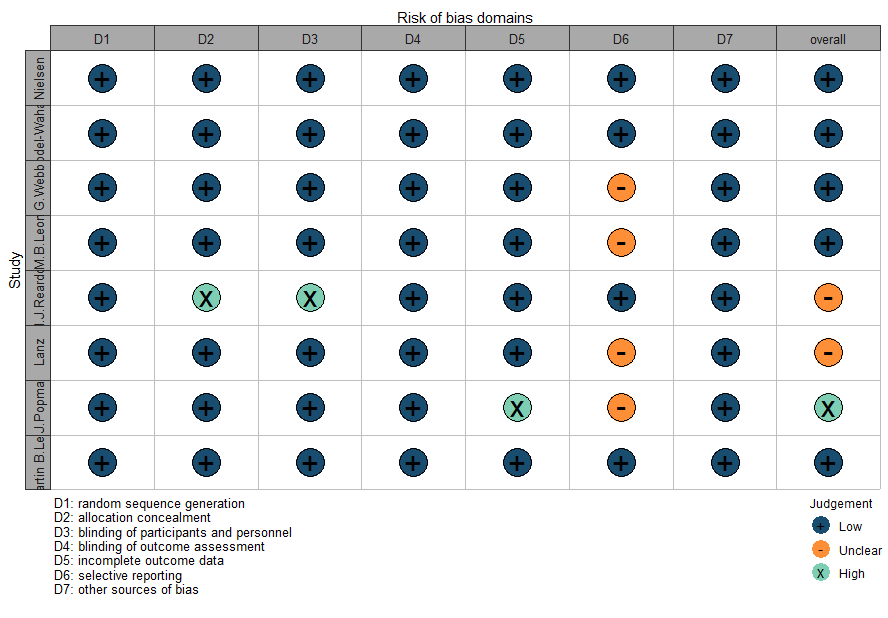

Supplement: Supplementary file 1 [file 2153-8174-26-7-36208-s1.zip › Supplementary Table 1-3.docx]
