# Supplementary material for: Incidence of Coronary Obstruction During Aortic Valve Implantation: Meta-Analysis and Mixt-Treatment Comparison of Self-Expandable Versus Balloon-Expandable Valve Prostheses
Source: Rev Cardiovasc Med. 2025 Jul 29;26(7):36208. doi: 10.31083/RCM36208 (PMC12326413; doi:10.31083/RCM36208)
Supplement: Supplementary file 1 [file 2153-8174-26-7-36208-s1.zip › Supplementary Table a-b.docx]

**Table a. Characteristics of studies excluded from the final analysis**

**(n=51)**

| Study | Year | Study design (Nr Centers) | Valve types | N= | Age (Years) | Sex (Female,%) | STS-score (%) | Logistic EuroSCORE | Intraoperative CO (%) | Approach for TAVI | CO in Approaches | Criteria | Analysis |
| --- | --- | --- | --- | --- | --- | --- | --- | --- | --- | --- | --- | --- | --- |
| Josep Rodés-Cabau et al. (1) | 2009 | Prospective  (6) | Cribier-Edwards; SAPIEN(BE); SAPIEN XT(BE) | 339 | 81±8 | 187(55.2) | 9.8±6.4 | NA | 3(0.9) | TF 49.3%  TA 50.7% | TA 1.1%  TF 0.6% | NA | Single rate |
| Thomas et al. (2) | 2010 | Prospective (32)  (SOURCE Cohort 1) | SAPIEN(BE) | 1,038 | 81.1±6.9 | 575(55.4) | NA | 27.6±15.5 | 6(0.58) | TF 44.6%  TA 55.4% | NA  NA | VARC | Single rate |
| Walther et al. (3) | 2011 | Prospective (3)  (TRAVERCE) | Cribier-Edwards; SAPIEN(BE) | 168 | 82.1±5.6 | 127(75.6) | NA | 27±12.7 | 6(3.6) | TA 100% | TA 3.6% | NA | Single rate |
| Nielsen et al.  (4) | 2012 | RCT (2)  (STACCATO) | SAPIEN(BE)  SAVR | 34  36 | 80.0±3.6  82.0±4.4 | 25(73.5)  24(66.7) | 3.1±1.5  3.4±1.2 | 9.4±3.9  10.3±5.8 | 2(5.9)  0 | TA 100%  \ | TA 5.9% | VARC | Single rate |
| Walther et al.  (5) | 2012 | Prospective (12) | SAPIEN XT(BE) | 150 | 81.6±5.8 | 61(40.7) | 7.5±4.4 | 24.3±7.0 | 0 | TA 98.7% | 0 | NA | Single rate |
| Treede et al.  (6) | 2012 | Prospective (7) | JenaValve(SE) | 67 | 83.1±3.9 | 32(47.8) | NA | 28.4±6.5 | 0 | TA 100% | 0 | NA | Single rate |
| J. Muñoz-García et al. (7) | 2013 | Prospective (42) | CoreValve(SE) | 1220 | 80.7±6.3 | 667(54.7) | NA | 17.8±13.0 | 8(0.7) | TF 94.7%  Subclavian 5.3% | NA  NA | VARC | Single rate |
| Watanabe et al. (8) | 2013 | PSM (2) | SAPIEN(BE)  CoreValve(SE) | 170  150 | 83.0±7.4  83.2±7.0 | 109(64.1)  67(44.7) | NA | 21.3±12.2  22.9±11.6 | 3(1.8)  0 | TF 100%  TF 100% | TF 1.8%  0 | VARC | Meta- and network meta-analysis |
| Abdel-Wahab et al. (9) | 2014 | RCT (5)  (CHOICE) | SAPIEN XT(BE)  CoreValve(SE) | 121  120 | 81.9  79.6 | 69(57.0)  86(71.7) | 5.6±2.9  6.2±3.9 | 21.5±12.9  22.1±14.7 | 2(1.6)  0 | TF 100%  TF 100% | TF 1.6%  0 | VARC | Meta- and network meta-analysis |
| Seiffert et al. (10) | 2014 | Prospective (1) | Engager(SE)  JenaValve(SE)  Acurate Symetis(SE) | 50  88  62 | 82.0±5.1  79.8±7.1  80.4±7.2 | 21(42.0)  26(29.5)  30(48.4) | 6.1±4.7  6.2±3.7  7.5±6.5 | 19.7±13.6  19.5±12.9  21.6±14.4 | 0  0  0 | TA 100%  TA 95.5%  TA 95.2% | 0  0  0 | VARC-2 | network meta analysis |
| Webb et al. (11) | 2014 | Prospective (16) | SAPIEN 3(BE) | 150 | 83.6±5.0 | 81(54.0) | 7.4±4.5 | 21.6±12.3 | 0 | TF 64%  TA 32.7%  T-aortic 3.3% | 0  0  0 | VARC-2 | Single rate |
| Maeda et al. (12) | 2015 | Prospective (1) | ACURATE neo(SE) | 15 | 83.3±6.0 | 11(73.3) | 7.5±3.1 | 21.9±10.6 | 0 | TF 66.7%  T-iliac 33.3% | 0  0 | VARC-2 | Single rate |
| G. Webb et al. (13) | 2015 | RCT  (28) | SAPIEN(BE)  SAPIEN XT(BE) | 276  284 | 84.6±8.6  84.1±8.7 | 134(48.6)  143(50.4) | 11.0±5.7  10.3±5.4 | 21.0±17.0  18.8±14.6 | 0  0 | TF 100%  TF 100% | 0  0 | VARC | network meta-analysis |
| Manoharan et al. (14) | 2015 | Prospective (6) | CoreValve Evolut R (SE) | 60 | 82.8±6.1 | 40(66.7) | 7.0±3.7 | 20.5±12.5 | 0 | TF 98.3%  T-aortic 1.7% | 0  0 | VARC-2 | Single rate |
| Schymik et al. (15) | 2015 | Prospective (99)  (SOURCE  XT) | SAPIEN XT(BE) | 2688 | 81.4±6.6 | 1550(57.7) | 7.9±6.6 | 20.4±12.4 | 12(0.4) | TF 62.7%  TA 33.3%  T-aortic 3.8%  Subclavian 0.2% | TF 0.4%  TA 0.4%  NA  NA | VARC | Single rate |
| Study | Year | Study design (Nr Centers) | Valve types | N= | Age (Years) | Sex (Female,%) | STS-score (%) | Logistic EuroSCORE | Intraoperative CO (%) | Approach for TAVI | CO in Approaches | Criteria | Analysis |
| G.P.Ussia et al. (16) | 2015 | Prospective (1) | CoreValve(SE) | 76 | 81.0±7.0 | 32(42.1) | 23.0±17.0 | 34.0±12.0 | 0 | TF 94.7%  T-axillary 5.3% | 0 | VARC-2 | Single rate |
| Linke et al. (17) | 2016 | Prospective  (12)  (Portico TAVI) | Portico(SE) | 222 | 83.0±4.6 | 165(74.3) | 5.8±3.3 | NA | 1(0.5) | TF 100% | TF 0.5% | VARC | Single rate |
| Bapat et al. (18) | 2016 | Prospective  (18)  (ROUTE Registry) | SAPIEN XT(BE) SAPIEN 3(BE) | 301 | 81.7±5.9 | 162(53.8) | 9.0±7.6 | 8.8±9.6 | 1(0.33) | T-aortic 100% | T-aortic 0.33% | VARC-2 | Single rate |
| Bocksch et al. (19) | 2016 | Prospective (1) | SAPIEN XT(BE)  SAPIEN 3(BE) | 102  107 | 82.0±0.5  81.4±0.6 | 68(66.7)  62(57.9) | NA  NA | 16.9±0.9  20.2±1.4 | 0 | TF 100%  TA 100% | 0  0 | VARC-2 | network meta-analysis |
| M.B.Leon et al. (20) | 2016 | RCT (57)  (PARTNER2 cohortA) | SAPIEN XT(BE)  SAVR | 1011  1021 | 81.5± 6.7  81.7± 6.7 | 463(45.8)  461(45.2) | 5.8±2.1  5.8±1.9 | NA  NA | 4(0.4)  6(0.6) | TF 76.7%  TA 17.2%  T-aortic 6.1%  \ | NA | VARC-2 | network meta-analysis |
| Vahanian et al. (21) | 2016 | Prospective (13) | SAPIEN 3(BE) | 101 | 84.4±3.8 | 55(54.5) | 5.2±1.7 | 13.2±3.8 | 0 | TF 100% | 0 | VARC-2 | Single rate |
| Petzina et al. (22) | 2016 | Prospective (1) | SAPIEN XT(BE)  SAPIEN 3(BE) | 53  46 | 82.0±5.3  81.8±6.2 | 33(62.3)  34(73.9) | 7.2±5.1  8.2±5.0 | 28.0±15.4  29.6±15.7 | 2(3.8)  0 | T-aortic 100%  T-aortic 100% | 2(3.8)  0 | VARC-2 | network meta-analysis |
| Kodali et al. (23) | 2016 | Prospective  (57)  (PARTNER2 SAPIEN 3) | SAPIEN 3(BE) | 1661 | 82.2±7.2 | 657(39.6) | 6.5±2.4 | NA | 5(0.3) | TF 86.9%  TA 8.3%  T-aortic 4.8% | NA | VARC-2 | Single rate |
| Silaschi et al. (24) | 2016 | Prospective  (15) | Jena Valve(SE) | 180 | 80.4±5.9 | 75(41.7) | 7.3±6.8 | 21.2±14.7 | 0 | TA 100% | 0 | VARC | Single rate |
| Jochen Wöhrle et al. (25) | 2016 | Prospective (1) | SAPIEN 3(BE) | 235 | 80.7±6.2 | 120(51.1) | 7.0±5.0 | 17.6±14.3 | 0 | TF 100% | 0 | VARC-2 | Single rate |
| Sung et al. (26) | 2016 | Prospective (11)  (Asian TAVR) | SAPIEN XT(BE)  Core Valve(SE) | 549  299 | 82.7±6.5  80.1±6.5 | 319(58.1)  133(44.5) | 5.4±3.8  5.0±3.8 | 16.4±11.2  16.6±13.2 | 8(1.5)  3(1.0) | TF 80.3%  TF 97.0% | NA  NA | VARC-2 | Meta- and network meta-analysis |
| Gonska et al. (27) | 2016 | Prospective (1) | SAPIEN 3(BE)  CoreValve(SE) | 100  100 | 81.7±6.4  80.7±5.9 | 51(51.0)  49(49.0) | 7.6±5.4  5.1±4.4 | NA  NA | 0  0 | TF 100%  TF 100% | 0  0 | VARC-2 | Meta- and network meta-analysis |
| Husser et al. (28) | 2017 | PSM (3) | ACURATE neo(SE)  SAPIEN 3(BE) | 311  622 | 81.0±6.0  81.0±6.0 | 189(60.8)  344(55.3) | NA  NA | 18.0±10.0  18.0±12.0 | 2(0.6)  0 | TF 100%  TF 100% | 2(0.6)  0 | VARC-2 | Meta- and network meta-analysis |
| Miura et al. (29) | 2017 | Prospective (1) | SAPIEN XT(BE) | 112 | 84.5±6.6 | 74(66.1) | 6.1±0.98 | 16.1±2.4 | 2(1.8) | TF 61.6%  TA 30.4%  T-iliac 8.0% | NA | VARC-2 | Single rate |
| J. Popma et al. (30) | 2017 | Prospective  (23) | CoreValve Evolut R(SE) | 241 | 83.3±7.2 | 165 (68.5) | 7.4±3.4 | NA | 1(0.4) | TF 89.5%  T-aortic 3.8%  Subclavian 6.7% | NA | VARC-2 | Single rate |
| M.J.Reardon et al. (31) | 2017 | RCT (87)  (SURTAVI) | CoreValve, Evolut R(SE)  SAVR | 863  794 | 79.9±6.2  79.7±6.1 | 366(42.4)  358(45.0) | 4.4±1.5  4.5±1.6 | 11.9±7.6  11.6±8.0 | 2(0.2)  0 | NA | NA | VARC-2 | network meta analysis |
| Wendler et al. (32) | 2017 | Prospective  (80)  (SOURCE 3) | SAPIEN 3(BE) | 1946 | 81.6±6.7 | 934(48.0) | NA | 18.3±13.2 | 7(0.4) | TF 87.1%  TA 9.3%  T-aortic 2.6%  T-arotid 0.6%  Subclavian 0.4% | TF 0.4%  NA | VARC-2 | Single rate |
| Study | Year | Study design (Nr Centers) | Valve types | N= | Age (Years) | Sex (Female,%) | STS-score (%) | Logistic EuroSCORE | Intraoperative CO (%) | Approach for TAVI | CO in Approaches | Criteria | Analysis |
| Silaschi et al. (33) | 2017 | Prospective  (15) | JenaValve(SE) | 30 | 74.4±9.3 | 18(60.0) | 4.9±3.5 | 17.7±14.8 | 0 | TA 100% | 0 | VARC | Single rate |
| Grube et al. (34) | 2017 | Prospective  (53)  (FORWARD) | CoreValve Evolut R(SE) | 1040 | 81.8±6.2 | 674(64.8) | 5.5±4.5 | 17.3±11.6 | 0 | TF 98%  TA 0.3%  T-carotid 0.1%  Subclavian 1.6% | 0 | VARC-2 | Single rate |
| Nakashima et al. (35) | 2017 | Prospective (1) | SAPIEN 3(BE)  SAPIEN XT(BE) | 54  112 | 83.3±1.5  85.9±1.2 | 42(78)  89(79) | 6.9±1.7  7.2±0.8 | 12.5±2.3  14.8±2.5 | 0  3(3) | TF 96.3% ;  T-aortic 1.9%  TF 72.3% ;  TA 25% | NA | VARC-2 | network meta analysis |
| Hellhammer et al. (36) | 2018 | PSM (1)  (prospective) | CoreValve Evolut R(SE)  CoreValve Evolut PRO(SE) | 148  74 | 81.2±5.6  81.4±4.5 | 110(74.3)  50(67.5) | NA  NA | 24.7±13.7  25.1±12.5 | 0  0 | TF 100%  TF 100% | 0  0 | VARC-2 | network meta analysis |
| Khaled Al-Shaibi et al. (37) | 2018 | Prospective (1) | SAPIEN XT; SAPIEN 3(BE) | 46 | 75.4±6.7 | 18(39.1) | 2.85±1.35 | NA | 2(4.35) | TF 91.3%  TA 8.7% | NA | NA | Single rate |
| Fischer et al. (38) | 2019 | Prospective (1) | SAPIEN XT(BE); SAPIEN 3(BE)  CoreValve(SE); CoreValve Evolut R(SE) | 275  227 | 81.5±8.3  81.2±9.9 | 109(39.6)  129(57.3) | NA  NA | 16.3±11.7  19.2±13.3 | 0  1(0.4) | TF 86.5%,TA 9.5%,  T-aortic 0.7%,  T-carotid 1.5%,  Brachiocephalic 1.8%  TF 83.3%,  T-aortic 0.4%,  T-carotid 1.8%,  subclavian 6.2%, Brachiocephalic 8.4% | NA | VARC-2 | Meta- and network meta-analysis |
| Lanz et al. (39) | 2019 | RCT (20)  (SCOPE I) | ACURATE neo(SE)  SAPIEN 3(BE) | 372  367 | 82.6±4.3  83.0±3.9 | 218(59)  202(55) | 3.7±0.41  3.4±0.44 | NA  NA | 0  0 | TF 100%  TF 100% | 0  0 | VARC-2 | Meta- and network meta-analysis |
| Pagnesi et al. (40) | 2019 | PSM (24)  (retrospective NEOPRO) | ACURATE neo(SE)  CoreValve Evolut PRO(SE) | 1263  288 | 81.8±5.8  81.7±5.9 | 819(64.8)  184(63.9) | 5.02±3.23  5.35±3.87 | NA  NA | 2(0.2)  1(0.4) | TF 100%  TF 100% | TF 0.2%  TF 0.4% | VARC-2 | network meta-analysis |
| J. Popma et al. (41) | 2019 | RCT (86)  (Evolut Low Risk) | CoreValve, Evolut R, Evolut PRO(SE)  SAVR | 725  678 | 74.1±5.8  73.6±5.9 | 261(36.0)  229(33.8) | 1.9±0.7  1.9±0.7 | NA  NA | 7(0.9)  3(0.4) | NA  \ | NA  \ | VARC-2 | network meta-analysis |
| Garrido et al. (42) | 2019 | Prospective  (10) | CoreValve(SE);  Portico(SE) | 384 | 82.2±1.7 | 202(52.6) | 5.9±3.7 | 14.3±5.3 | 3(0.8) | NA | NA | NA | Single rate |
| Rodés-Cabau et al. (43) | 2019 | Prospective (4)  (RADIANT) | HLT Meridian valve(SE) | 25 | 85.0±6.0 | 5(20.0) | 5.1±3.2 | NA | 0 | TF 100% | 0 | VARC-2 | Single rate |
| Tchétché et al. (44) | 2019 | Prospective  (23)  (VIVA Trail) | CoreValve; Evolut R(SE) | 202 | 79.9±7.2 | 106(52.5) | 6.6±5.1 | 25.0±14.3 | 0 | TF 96.5%  T-aortic 0.5%  T-carotid 1.0% | NA | VARC-2 | Single rate |
| Won-Keun Kim et al. (45) | 2020 | Retrospective  (1) | ACURATE neo(SE) | 1000 | 81.9±1.0 | 660(66.0) | NA | 19.3±2.3 | 0 | TF 100% | 0 | VARC-2 | Single rate |
| Manoharan et al. (46) | 2020 | Prospective  (39) | CoreValve Evolut PRO(SE) | 629 | 81.7±6.1 | 389(61.8) | 4.7±3.3 | NA | 1 (0.2) | TF 97% | NA | VARC-2 | Single rate |
| Saia et al. (47) | 2020 | Prospective(9) | SAPIEN 3 Ultra(BE) | 139 | 81.4±8.3 | 77(55.4) | 3.8±2.4 | 14.0±11.5 | 0 | TF 100% | 0 | VARC-2 | Single rate |
| Ulrich Schäfer et al. (48) | 2020 | Prospective  (11)  (BIOVALVE) | Biovalve(SE) | 68 | 82.4±5.3 | 42(61.8) | 5.3±3.6 | 14.2±7.2 | 0 | TF 100% | 0 | VARC-2 | Single rate |
| Study | Year | Study design (Nr Centers) | Valve types | N= | Age (Years) | Sex (Female,%) | STS-score (%) | Logistic EuroSCORE | Intraoperative CO (%) | Approach for TAVI | CO in Approaches | Criteria | Analysis |
| Yong et al. (49) | 2020 | Prospective  (11) | SAPIEN XT(BE) | 199 | 85.5±4.51 | 108 (54.3) | 5.9±2.59 | 18.5±11.19 | 0 | TF 100% | 0 | VARC-2 | Single rate |
| John K. Forrest et al. (50) | 2020 | Prospective  (25) | CoreValve Evolut R(SE);  Evolut PRO(SE) | 150 | 70.3±5.5 | 72(48) | 1.4±0.6 | NA | 1(0.7) | TF 98.7%  Subclavian 1.3% | NA | VARC-2 | Single rate |
| Martin B. Leon et al. (51) | 2021 | RCT (71)  (PARTNER 3) | SAPIEN 3(BE)  SAVR | 496  454 | 73.3±5.8  73.6±6.1 | 161(32.5)  131(28.9) | 1.9±0.7  1.9±0.6 | NA  NA | 1(0.2)  2(0.4) | TF 100%  \ | TF 0.2%  SAVR 0.4% | VARC-2 | network meta-analysis |

Values are n (%). SEV = Self-Expandable valves; BE = Balloon-Expandable valves; TF = Trans-femoral; TS = Trans-subclavian; TA = Trans-apical; T-iliac = Trans-iliac; T-aortic = Trans-aortic, T- carotid = Trans-carotid; T-axillary = Trans-axillary

**Table b. Table summarizing CO rate in the included studies**

|  | **Studies** | **CO** | **Cohort patients** | **Average CO rate (%)** | **Min (%)** | **Max (%)** |
| --- | --- | --- | --- | --- | --- | --- |
| ***Mechanisms*** |  |  |  |  |  |  |
| BEV | 29 | 69 | 14052 | 0.49 | 0 | 5.9 |
| SEV | 29 | 33 | 10749 | 0.31 | 0 | 1.0 |
| SAVR | 5 | 11 | 2983 | 0.37 | 0 | 0.6 |
| ***Valves*** |  |  |  |  |  |  |
| SAPIEN | 3 | 5 | 480 | 1.04 | 0 | 5.9 |
| SAPIEN XT | 8 | 19 | 2295 | 0.83 | 0 | 3.8 |
| SAPIEN 3 | 8 | 1 | 2004 | 0.05 | 0 | 0.2 |
| CoreValve | 8 | 7 | 1569 | 0.45 | 0 | 3.8 |
| Evolut R | 5 | 8 | 1103 | 0.73 | 0 | 2.2 |
| Evolut PRO | 3 | 3 | 524 | 0.57 | 0 | 1.2 |
| ACURATE neo | 3 | 4 | 1946 | 0.21 | 0 | 0.6 |
| SAVR | 5 | 11 | 2983 | 0.37 | 0 | 0.6 |
