# Supplementary figures and images for: Incidence of Coronary Obstruction During Aortic Valve Implantation: Meta-Analysis and Mixt-Treatment Comparison of Self-Expandable Versus Balloon-Expandable Valve Prostheses
Source: Rev Cardiovasc Med. 2025 Jul 29;26(7):36208. doi: 10.31083/RCM36208 (PMC12326413; doi:10.31083/RCM36208)

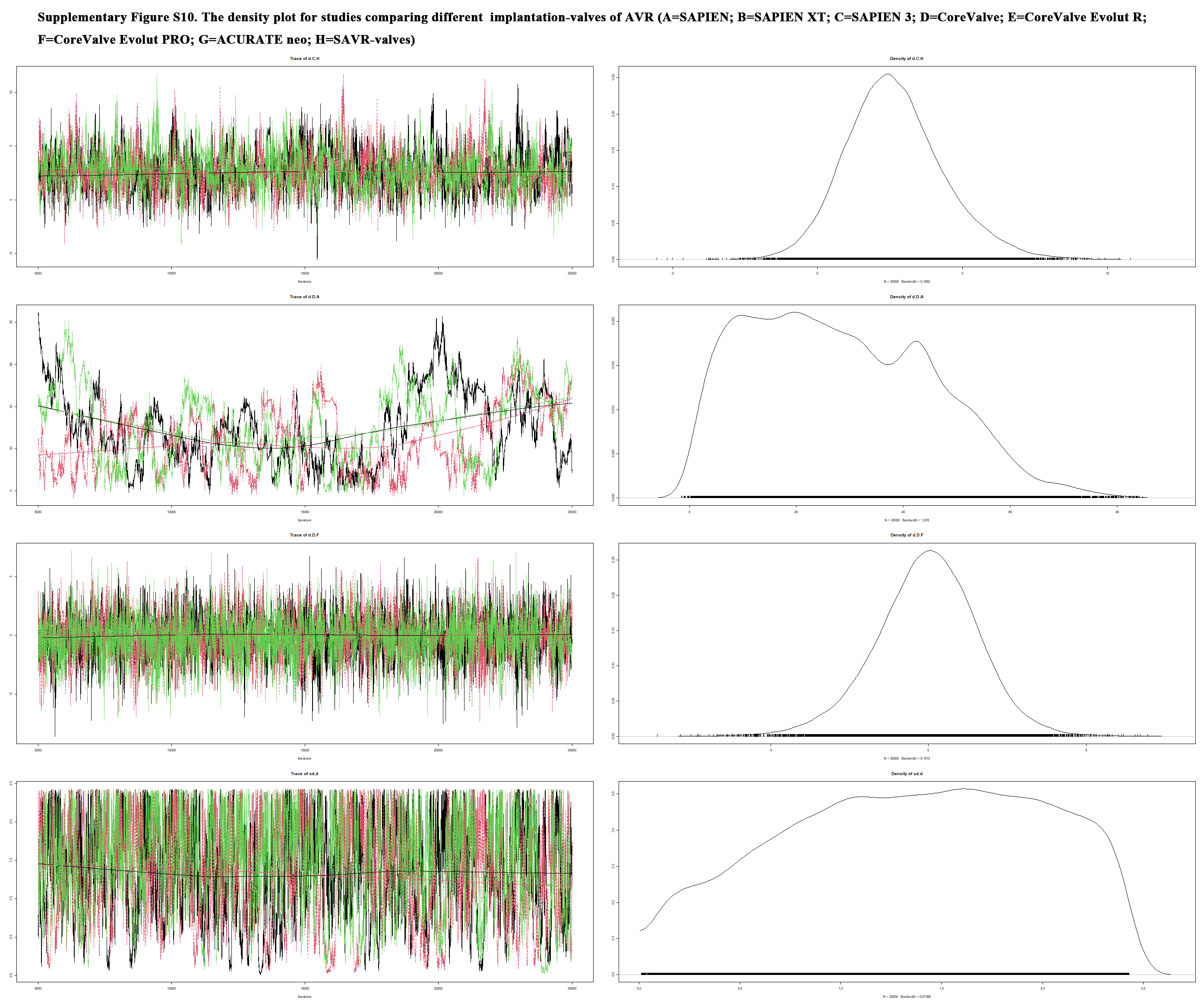

Supplement: Supplementary file 1 [file 2153-8174-26-7-36208-s1.zip › Supplementary figures/Supplementary Fig. 10.tiff]

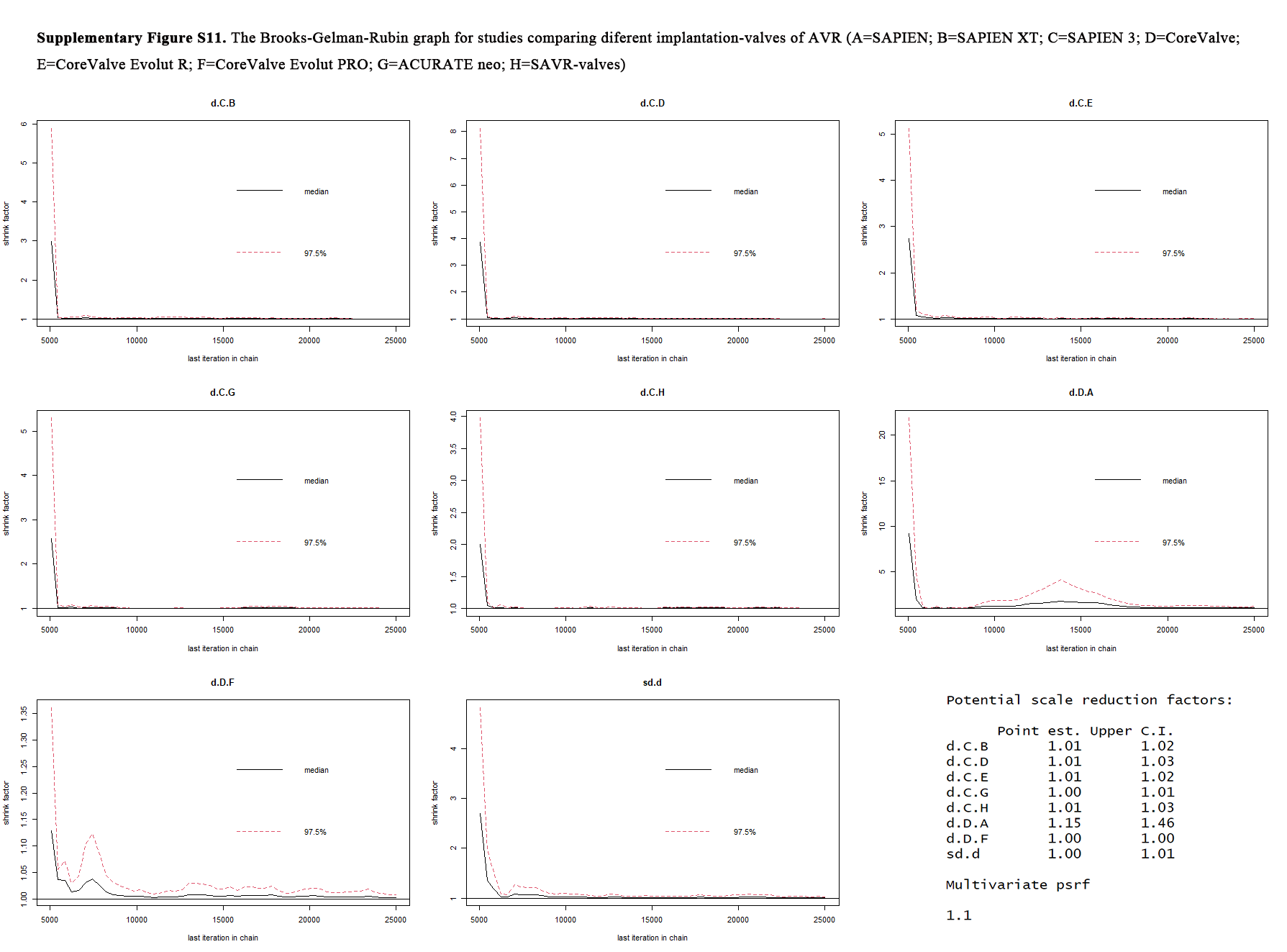

Supplement: Supplementary file 1 [file 2153-8174-26-7-36208-s1.zip › Supplementary figures/Supplementary Fig. 11.tiff]

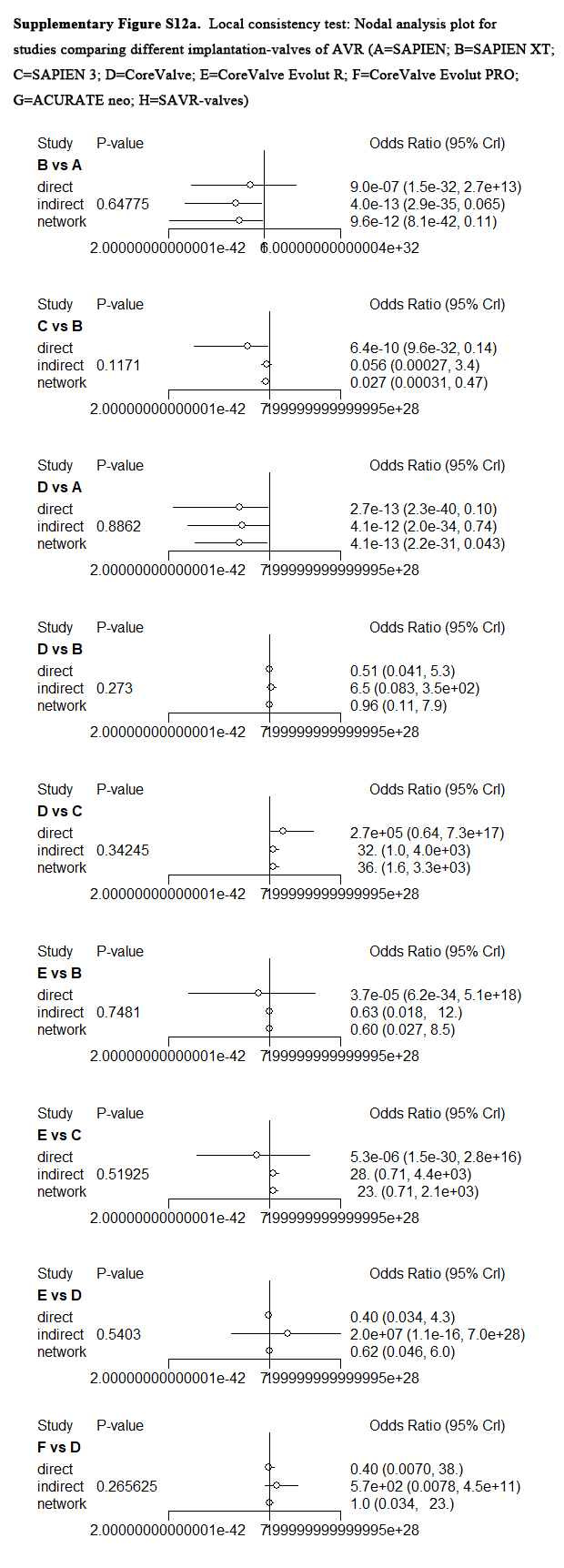

Supplement: Supplementary file 1 [file 2153-8174-26-7-36208-s1.zip › Supplementary figures/Supplementary Fig. 12a.tiff]

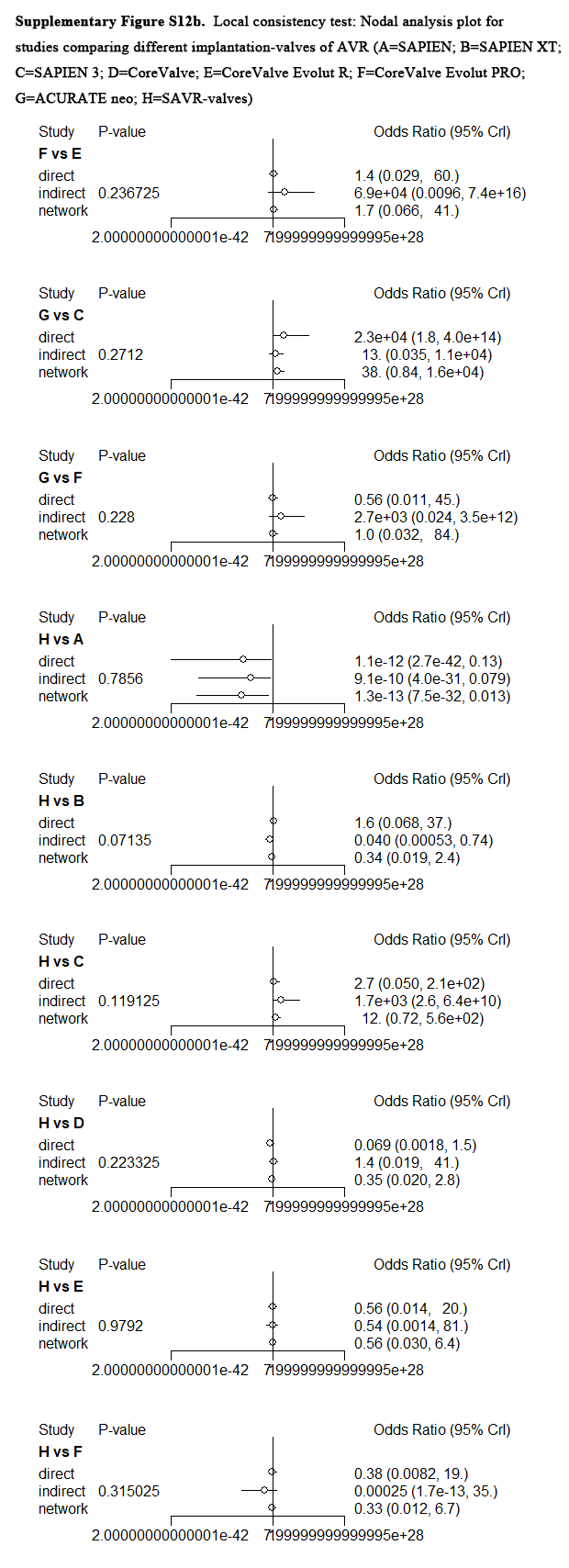

Supplement: Supplementary file 1 [file 2153-8174-26-7-36208-s1.zip › Supplementary figures/Supplementary Fig. 12b.tiff]

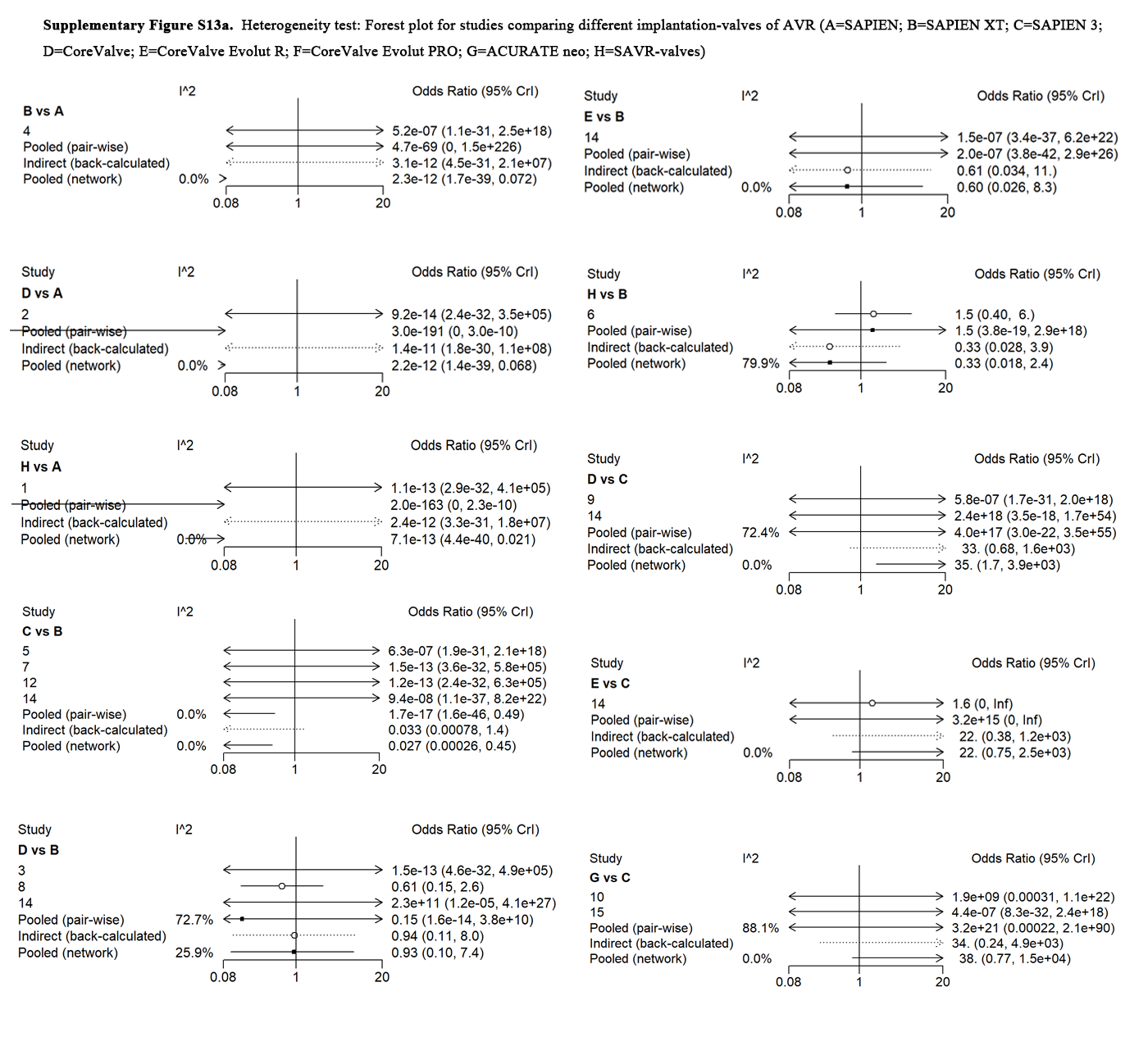

Supplement: Supplementary file 1 [file 2153-8174-26-7-36208-s1.zip › Supplementary figures/Supplementary Fig. 13a.tiff]

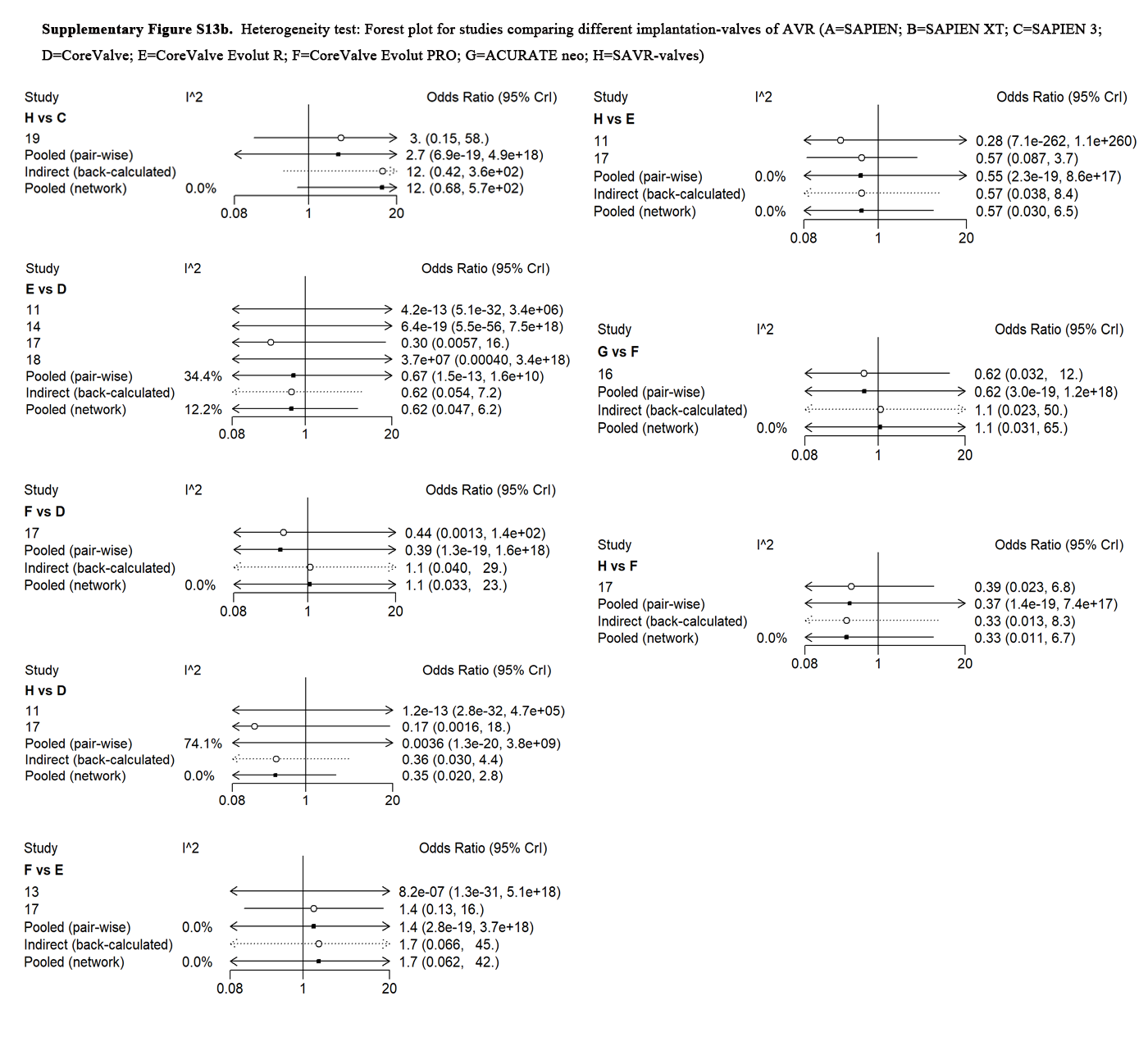

Supplement: Supplementary file 1 [file 2153-8174-26-7-36208-s1.zip › Supplementary figures/Supplementary Fig. 13b.tiff]

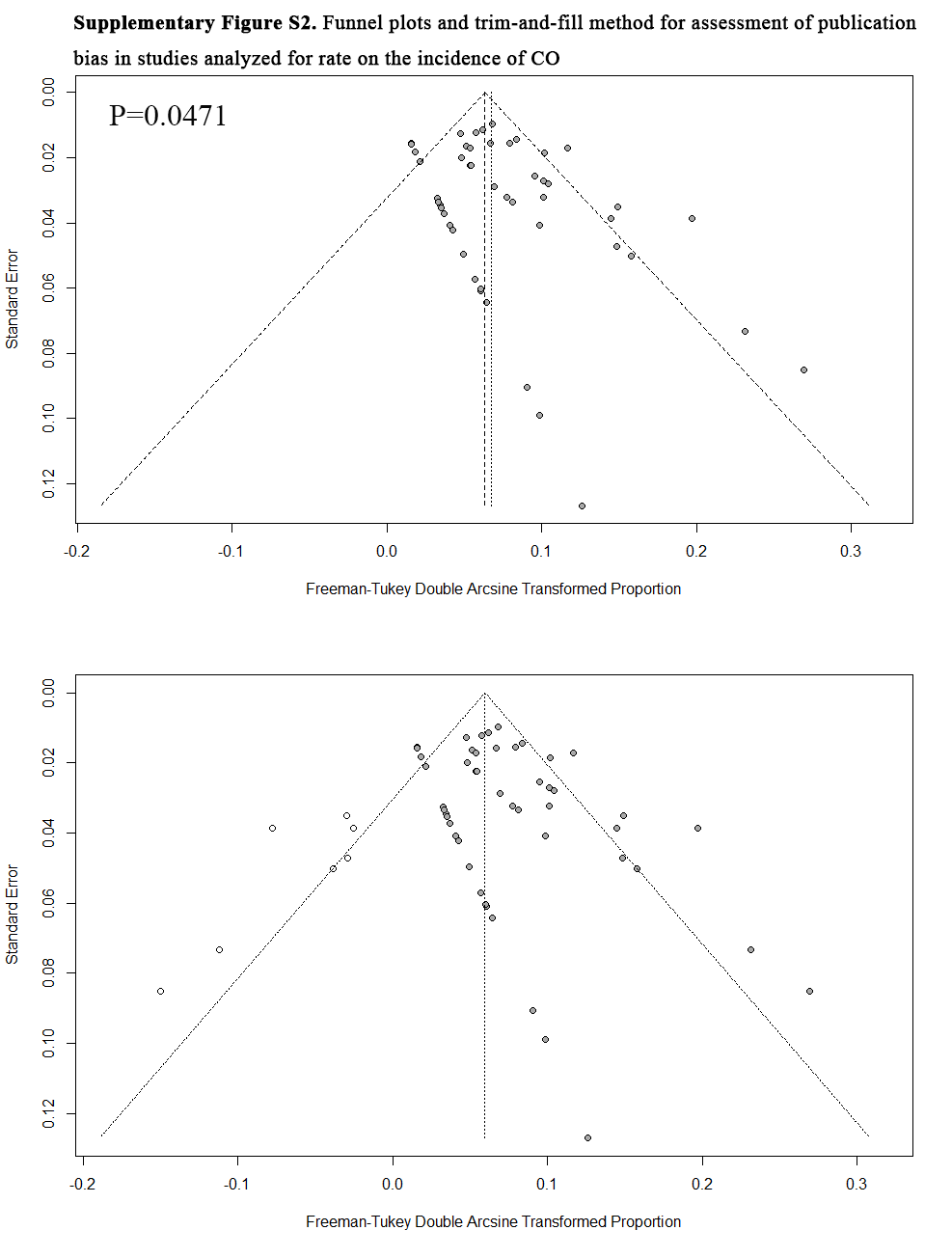

Supplement: Supplementary file 1 [file 2153-8174-26-7-36208-s1.zip › Supplementary figures/Supplementary Fig. 2.tiff]

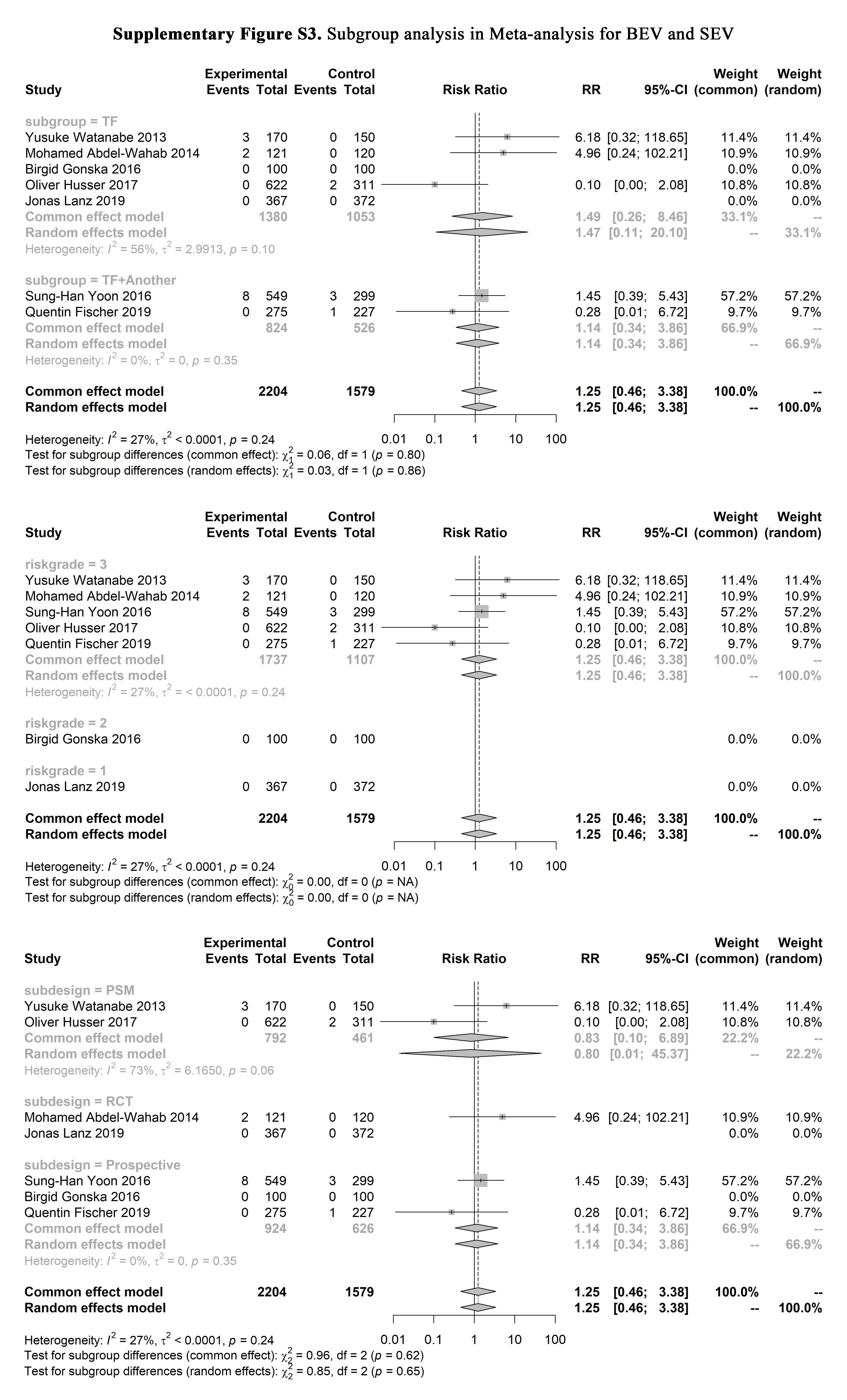

Supplement: Supplementary file 1 [file 2153-8174-26-7-36208-s1.zip › Supplementary figures/Supplementary Fig. 3.tiff]

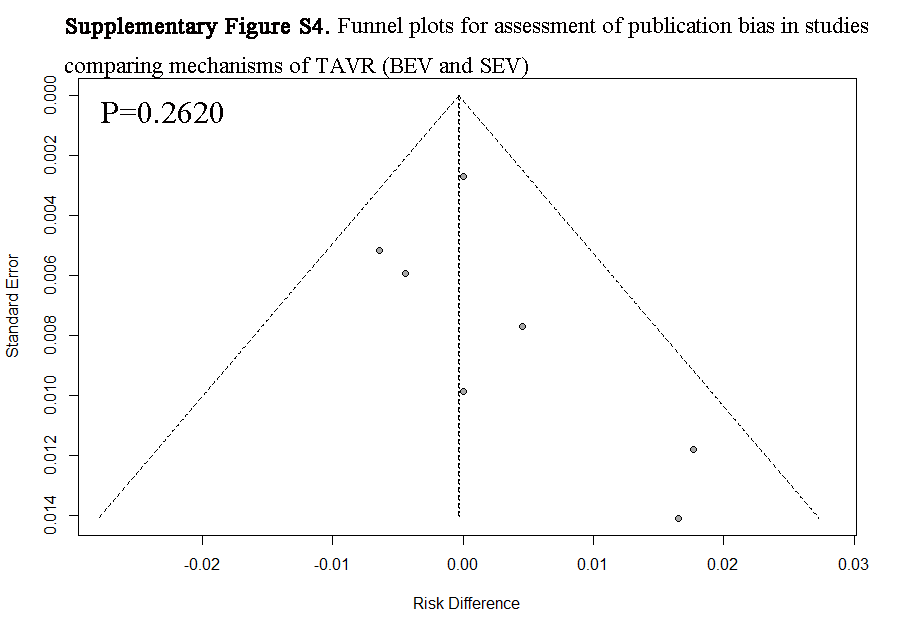

Supplement: Supplementary file 1 [file 2153-8174-26-7-36208-s1.zip › Supplementary figures/Supplementary Fig. 4.tiff]

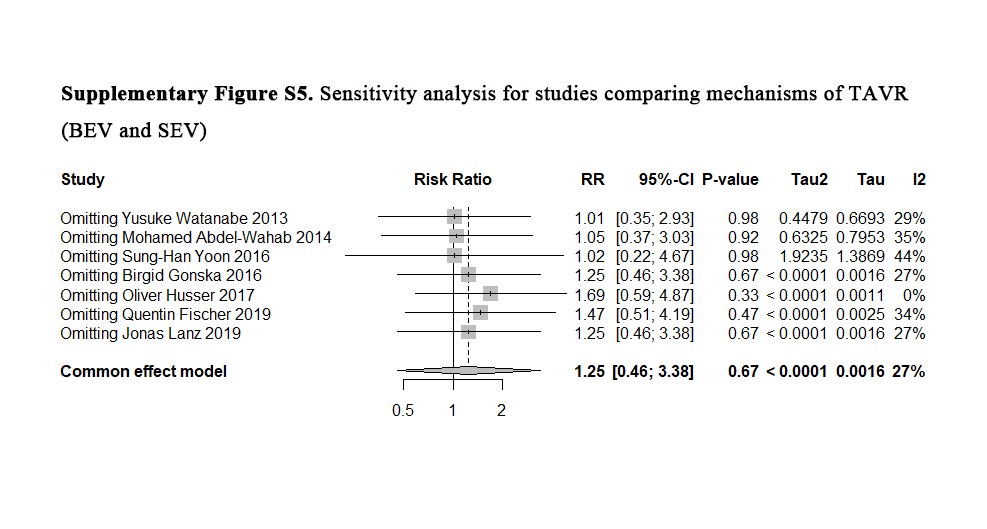

Supplement: Supplementary file 1 [file 2153-8174-26-7-36208-s1.zip › Supplementary figures/Supplementary Fig. 5.tiff]

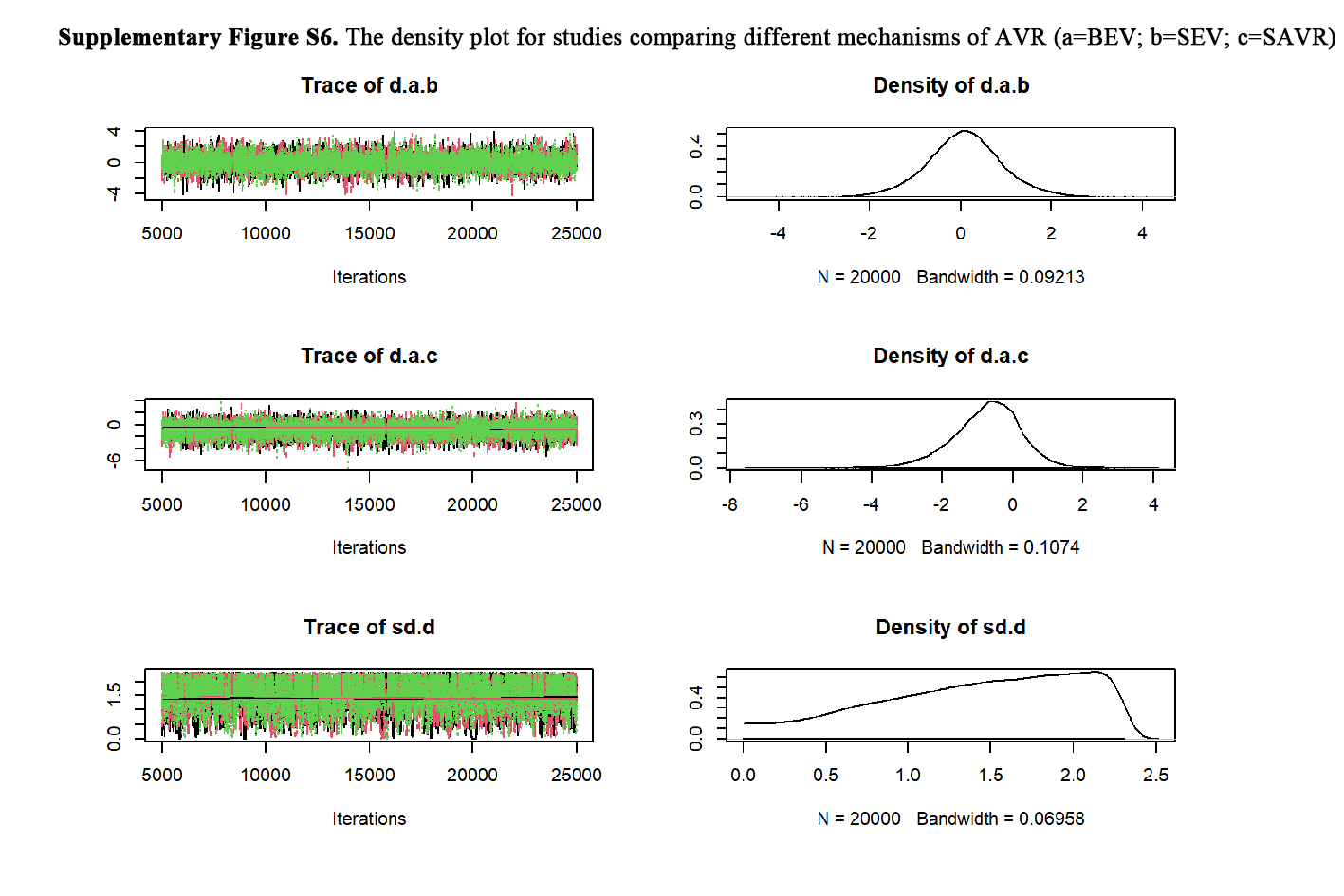

Supplement: Supplementary file 1 [file 2153-8174-26-7-36208-s1.zip › Supplementary figures/Supplementary Fig. 6.tiff]

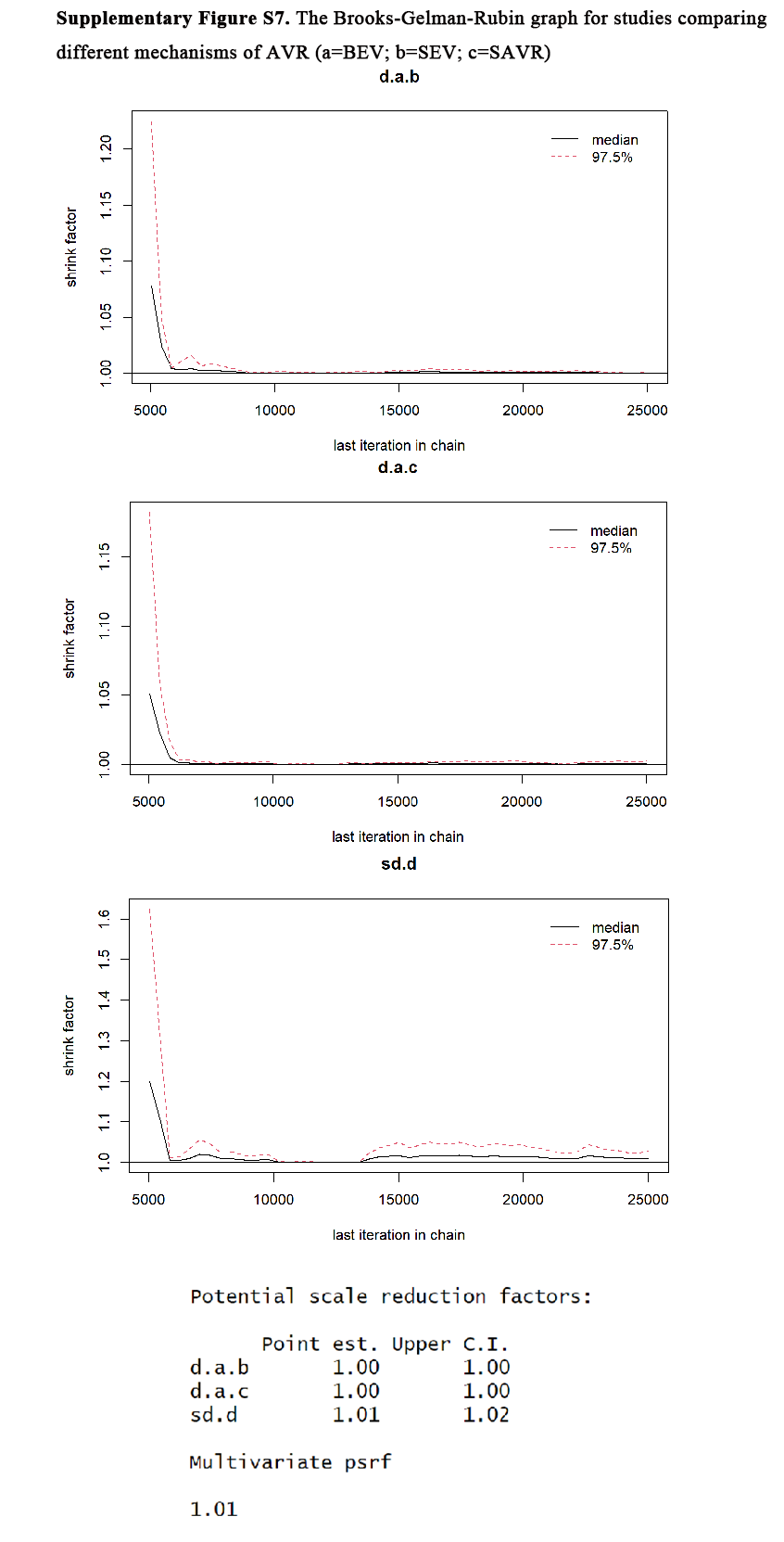

Supplement: Supplementary file 1 [file 2153-8174-26-7-36208-s1.zip › Supplementary figures/Supplementary Fig. 7.tiff]

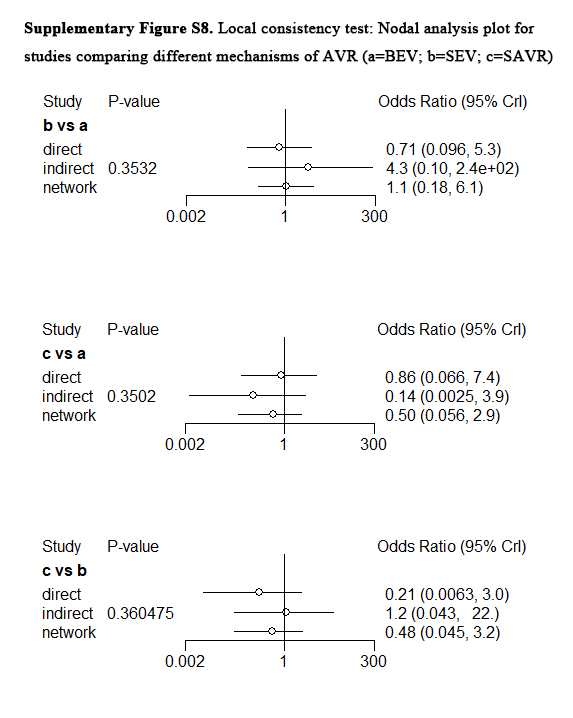

Supplement: Supplementary file 1 [file 2153-8174-26-7-36208-s1.zip › Supplementary figures/Supplementary Fig. 8.tiff]

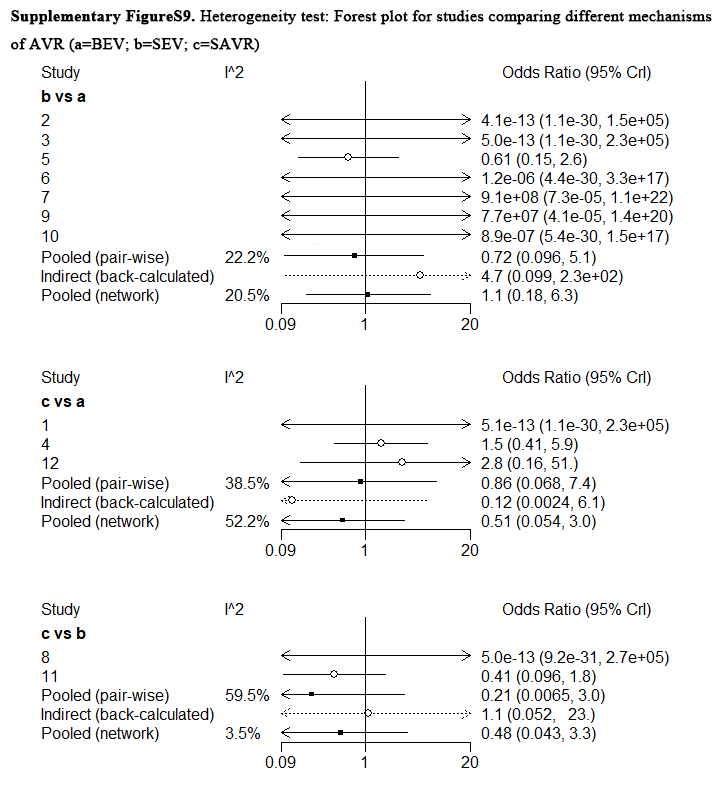

Supplement: Supplementary file 1 [file 2153-8174-26-7-36208-s1.zip › Supplementary figures/Supplementary Fig. 9.tiff]
